# Division of labor within the DNA damage tolerance system reveals non-epistatic and clinically actionable targets for precision cancer medicine

**DOI:** 10.1093/nar/gkac545

**Published:** 2022-07-12

**Authors:** Aldo Spanjaard, Ronak Shah, Daniël de Groot, Olimpia Alessandra Buoninfante, Ben Morris, Cor Lieftink, Colin Pritchard, Lisa M Zürcher, Shirley Ormel, Joyce J I Catsman, Renske de Korte-Grimmerink, Bjørn Siteur, Natalie Proost, Terry Boadum, Marieke van de Ven, Ji-Ying Song, Maaike Kreft, Paul C M van den Berk, Roderick L Beijersbergen, Heinz Jacobs

**Affiliations:** Division of Tumor Biology and Immunology, The Netherlands Cancer Institute, Plesmanlaan 121, 1066 CX Amsterdam, The Netherlands; Division of Tumor Biology and Immunology, The Netherlands Cancer Institute, Plesmanlaan 121, 1066 CX Amsterdam, The Netherlands; Division of Tumor Biology and Immunology, The Netherlands Cancer Institute, Plesmanlaan 121, 1066 CX Amsterdam, The Netherlands; Division of Tumor Biology and Immunology, The Netherlands Cancer Institute, Plesmanlaan 121, 1066 CX Amsterdam, The Netherlands; Division of Molecular Carcinogenesis, The Netherlands Cancer Institute, Plesmanlaan 121, 1066 CX Amsterdam, The Netherlands; Division of Molecular Carcinogenesis, The Netherlands Cancer Institute, Plesmanlaan 121, 1066 CX Amsterdam, The Netherlands; Intervention unit of the Mouse Clinic for Cancer and Aging research (MCCA), The Netherlands Cancer Institute, Plesmanlaan 121, 1066 CX Amsterdam, The Netherlands; Division of Tumor Biology and Immunology, The Netherlands Cancer Institute, Plesmanlaan 121, 1066 CX Amsterdam, The Netherlands; Division of Tumor Biology and Immunology, The Netherlands Cancer Institute, Plesmanlaan 121, 1066 CX Amsterdam, The Netherlands; Division of Tumor Biology and Immunology, The Netherlands Cancer Institute, Plesmanlaan 121, 1066 CX Amsterdam, The Netherlands; Intervention unit of the Mouse Clinic for Cancer and Aging research (MCCA), The Netherlands Cancer Institute, Plesmanlaan 121, 1066 CX Amsterdam, The Netherlands; Intervention unit of the Mouse Clinic for Cancer and Aging research (MCCA), The Netherlands Cancer Institute, Plesmanlaan 121, 1066 CX Amsterdam, The Netherlands; Intervention unit of the Mouse Clinic for Cancer and Aging research (MCCA), The Netherlands Cancer Institute, Plesmanlaan 121, 1066 CX Amsterdam, The Netherlands; NKI Animal facility, The Netherlands Cancer Institute, Plesmanlaan 121, 1066 CX Amsterdam, The Netherlands; Intervention unit of the Mouse Clinic for Cancer and Aging research (MCCA), The Netherlands Cancer Institute, Plesmanlaan 121, 1066 CX Amsterdam, The Netherlands; Division of Experimental Animal Pathology, The Netherlands Cancer Institute, Plesmanlaan 121, 1066 CX Amsterdam, The Netherlands; Division of Tumor Biology and Immunology, The Netherlands Cancer Institute, Plesmanlaan 121, 1066 CX Amsterdam, The Netherlands; Division of Tumor Biology and Immunology, The Netherlands Cancer Institute, Plesmanlaan 121, 1066 CX Amsterdam, The Netherlands; Division of Molecular Carcinogenesis, The Netherlands Cancer Institute, Plesmanlaan 121, 1066 CX Amsterdam, The Netherlands; Division of Tumor Biology and Immunology, The Netherlands Cancer Institute, Plesmanlaan 121, 1066 CX Amsterdam, The Netherlands

## Abstract

Crosslink repair depends on the Fanconi anemia pathway and translesion synthesis polymerases that replicate over unhooked crosslinks. Translesion synthesis is regulated via ubiquitination of PCNA, and independently via translesion synthesis polymerase REV1. The division of labor between PCNA-ubiquitination and REV1 in interstrand crosslink repair is unclear. Inhibition of either of these pathways has been proposed as a strategy to increase cytotoxicity of platinating agents in cancer treatment. Here, we defined the importance of PCNA-ubiquitination and REV1 for DNA in mammalian ICL repair. In mice, loss of PCNA-ubiquitination, but not REV1, resulted in germ cell defects and hypersensitivity to cisplatin. Loss of PCNA-ubiquitination, but not REV1 sensitized mammalian cancer cell lines to cisplatin. We identify polymerase Kappa as essential in tolerating DNA damage-induced lesions, in particular cisplatin lesions. *Polk-*deficient tumors were controlled by cisplatin treatment and it significantly delayed tumor outgrowth and increased overall survival of tumor bearing mice. Our results indicate that PCNA-ubiquitination and REV1 play distinct roles in DNA damage tolerance. Moreover, our results highlight POLK as a critical TLS polymerase in tolerating multiple genotoxic lesions, including cisplatin lesions. The relative frequent loss of *Polk* in cancers indicates an exploitable vulnerability for precision cancer medicine.

## Graphical Abstract

**Graphical Abstract:**
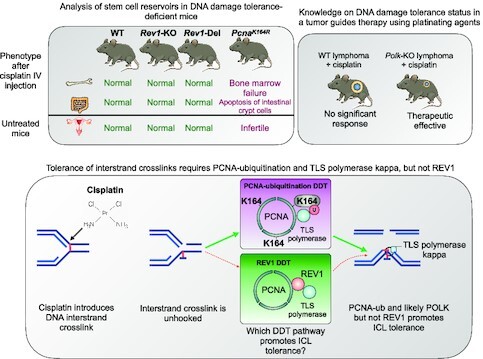
Human and murine cell lines and mice rely on PCNA-ubiquitination, though not REV1, to tolerate cisplatin-induced DNA interstrand crosslinks. TLS polymerase Kappa deficiency sensitises tumors to cisplatin *in vitro* and *in vivo*.

## INTRODUCTION

Platinating agents, such as cisplatin, are mainstays in the treatment of a multitude of solid and hematological malignancies (1). Cisplatin induces highly toxic DNA interstrand crosslinks (ICLs), which block replication and transcription by preventing the separation of the DNA strands. These ICLs are normally repaired via the Fanconi anemia (FA) pathway (2). In humans, absence of the FA repair pathway results in the cancer predisposition FA syndrome that is characterized by bone marrow failure, depletion of hematopoietic stem cells (HSCs), reduced fertility, and increased incidence of cancer (3,4). Importantly, FA-patients are highly sensitive to drugs that induce ICLs, such as cisplatin (4).

FA-mediated resolution of ICLs requires that incisions are made on one strand in the DNA sugar-phosphate backbone adjacent to the crosslink, which enables its unhooking. The unhooked ICL lesion stalls high-fidelity replicative DNA polymerases (5), and requires specialized error-prone translesion synthesis (TLS) to continue replication opposite the lesion. In this way, TLS provides the template for subsequent homology directed repair that is required in ICL. In the absence of TLS, ICL repair is severely diminished (5), and cells are greatly sensitized to various types of ICL-inducing agents (6).

A central pathway of TLS is promoted by site-specific mono-ubiquitination of the DNA sliding clamp Proliferating Cell Nuclear Antigen (PCNA-mUb) on lysine residue 164 (K164), mediated by the E2-E3 ubiquitin conjugase/ligase complex Rad6/Rad18 (7,8). Additionally, the TLS polymerase REV1 can bypass lesions independently of PCNA-mUb, and has a unique capacity to recruit other TLS polymerases via its specific C-terminal domain (9,10).

We have previously shown *in vivo* that injection of cisplatin resulted in complete bone marrow failure and hematopoietic stem cell depletion in *Pcna^K164R/K164R^* mice (from here on termed *Pcna^K164R^*), in which PCNA-ubiquitination is abolished (6). Others have shown that *Rev1*-deficient mice developed progressive bone marrow failure in compound mutants, which besides *Rev1* lack *Xpc* and therefore are also deficient for global nucleotide excision repair (NER) (11), probably due to endogenous lesions. However, the extent to which REV1 is required to tolerate cisplatin-induced lesions *in vivo* is not known. Moreover, both inhibition of PCNA- or REV1-dependent DNA damage tolerance (DDT) have been proposed to chemosensitize cancers to cisplatin (12,13), enhancing the efficacy of cancer treatment with platinating agents. However, it is unclear if, and to what extent, PCNA-ubiquitination or REV1 can compensate for the absence of each other in processing unhooked cisplatin ICLs. Moreover, because PCNA-ubiquitination and REV1 can recruit various distinct TLS polymerases, it is likely that loss of specific TLS polymerases sensitizes tumors to cisplatin. However, which TLS polymerase participates in ICL repair and whether its loss in tumors enhances the efficacy of cisplatin treatment remains to be examined.

Here, we studied the relevance of PCNA-ubiquitination and REV1 in the bypass of cisplatin lesions *in vivo* and *in vitro*. We generated *Rev1-*deficient mice, and compared them to *Pcna^K164R^* mice. We report that PCNA-ubiquitination, but strikingly not REV1, is essential in bypassing cisplatin lesions *in vivo*. Moreover, REV1 is dispensable for the tolerance of cisplatin lesions in various murine and human cancer cells *in vitro*. Interestingly, we identify TLS polymerase Kappa (POLK) to be essential for tolerance of cisplatin lesions in a murine lymphoma model. High-throughput compound screens revealed POLK to be essential to tolerate various classes of DNA damage-induced lesions. *In vivo*, cisplatin treatment significantly delayed the outgrowth of POLK-deficient lymphomas, and substantially increased the overall survival time of tumor bearing mice.

## MATERIALS AND METHODS

### Reagents, strains, antibody-usage and PCR settings

See supplementary Tables S5-S12 for extensive details.

### Mouse experiments

All mice were kept on C57BL/6J background under specific pathogen-free conditions. The *Pcna^K164R^* knock-in mouse model has been described previously (14). To generate the *Rev1*-KO model, zygotes isolated from C57BL/6N mice were co-injected with *in vitro* transcribed Cas9 mRNAs and sgRNAs (target sequences 5'-AGAAATCTAATGATGTTGCATGG-3' and 5'-TGAAGCACTGATTGACGTCACGG-3', designed using the crispr.mit.edu tool). The first sgRNA targets Cas9 to exon 4 of the *Rev1* locus (ENSMUSE00000155622) and the second sgRNA targets Cas9 to exon 11 of the *Rev1* locus (ENSMUSE00000155613). Offspring was tested by PCR for *Rev1* inactivating mutations using the primers (FW: 5'-GGCAACATGGCCAAGAAGAAC-3', RV1: 5'-ACTCAGTCAGCAGACACATGC-3', RV2: 5'-TTATTCAGCTTGGCGAGCGCTTTTG-3') and ABI sequencing (3730 DNA analyzer, Applied Biosystems). A *Rev1* mutant, carrying a 4800 bp deletion (1514 bp in cDNA) was selected, backcrossed three times onto C57BL/6J and maintained heterozygous. A second C57BL/6J *Rev1*-deficient strain was generated using the Cre-Lox system: LoxP-flanked sites were introduced in the *Rev1* locus in the intron before exon 4 and after exon 13, using CRISPR/Cas9 and LoxP containing HR template to the selected sites. sgRNA sequences were: 5′-CCCTAGCCCTTTAATATAACAGG-3′, 5′-CAAACGTGCATTCGAGGGACAGG-3′ and LoxP sequences were: 5′-ACCAAAGTTTCCTCTGAGTCAGTGATTGTCAACCCTCTTAATGCCCTAGCCCTTTAATATgacctaATAACTTCGTATAGCATACATTATACGAAGTTATATtaagggttAACAGGTCCTTATGATATGGTGACCCCAACAATAAAATTATTTTTGTTGTACTTCATAAC-3′ and 5′-CCACAGGTTGAGATACCCTGCCTCTGTAGCAGTAATGCAAGTGAATTGGTGTTTCCTGTCgacctaATAACTTCGTATAGCATACATTATACGAAGTTATATtaagggttCCTCGAATGCACGTTTGCATTGGGCACCTCTCTGGGATTTAGGGCCATACACTGCACATT-3′. These mice were crossed with Actin-Cre mice to obtain the mice containing a deletion of *Rev1*, termed *Rev1*-Del mice, after which these mice were crossed with C57BL/6J to exclude the Actin-Cre transgene as a potential confounder. For genotyping, the wild-type and mutant alleles are be distinguished by size differences of PCR products. Experiments were approved by an independent animal ethics committee of the Netherlands Cancer Institute (Amsterdam, Netherlands) and executed according national and European guidelines.

### Histopathological analysis

For histopathological analysis, organs were collected and fixed in ethanol-acetic acid-formaldehyde (EAF) for 24 h after necropsy. Fixed tissues and organs were subsequently dehydrated, embedded in paraffin, and 2 μm sections were prepared and stained with hematoxylin and eosin (H&E).

### Generation of mouse embryonic fibroblast (MEFs) and PreB cells

PreB cells and MEFs were isolated from time-mated intercrosses of WT, *Pcna^K164R^* and *Rev1*-KO mice using standard procedures. For *Rev1-*Del mice, only MEFs were isolated. For the generation of PreB cell cultures, E14.5 embryos were isolated and single cell suspensions were generated from fetal livers and subsequently cultured on ST2 feeder cells in IL7-containing complete medium (Iscoves, 8% Fetal Calf Serum (FCS) and Penicillin and Streptomycin (Pen/Strep)) (15). MEFs were isolated as described previously (16). MEFs were immortalized using lentiviral expression of a p53-specific shRNA (17).

### Cell culture

p53-KO lymphomas have been described previously (18). p53-KO lymphomas were cultured in RPMI 1640 supplemented with 8% FCS, Pen/Strep, L-asparagine, and ß-Mercaptoethanol. MEFs and PreB cells were cultured in IMDM, supplemented with 8% FCS, Pen/Strep and ß-mercaptoethanol. Additionally, PreB medium was supplemented with IL-7 containing medium, and PreB cells were grown on irradiated ST2 feeder cells as described above). LNCaP, and 22RV1 human prostate cancer cells were grown in RPMI 1640, supplemented with 8% FCS and Pen/Strep. MCF-7 (19) and HCT116 (20) p53-WT and p53-KO lines have been described previously. HCT116 was grown in RPMI 1640 supplemented with 10% FCS and Pen/Strep. MCF-7 was grown in DMEM, supplemented with 8% FCS, Pen/Strep and GlutaMax. HEK293T cells were grown in DMEM, supplemented with 8% FCS, and Pen/Strep.

### Somatic hypermutation analysis

DNA was extracted from germinal center B cells isolated from Peyer’s patches using proteinase K treatment and ethanol precipitation. The JH4 flanking intronic sequence of endogenous rearrangements of VHJ558 family members were amplified during 40 cycles of PCR using PFU Ultra polymerase (Stratagene), as described previously (21). PCR products were purified using the QIAquick Gel Extraction kit (Qiagen) and cloned into the TOPO II blunt vector (Invitrogen Life Technologies) and sequenced on a 3730 DNA analyzer (Applied Biosystems). Sequence alignment was performed using Seqman software (DNAStar). Calculations exclude non-mutated sequences, insertions and deletions. Clonal sequences were counted only once.

### *In vitro* class switch recombination assay

Spleens were isolated from WT and *Rev1*-KO mice. Single cell suspensions were generated and subjected to erythrocyte lysis. Naïve splenic B cells were enriched by performing a CD43 depletion using a biotinylated anti-CD43 antibody, followed by Streptavidin magnetic bead incubation. Cells were subsequently stimulated with Lipopolysaccharide (*Escherichia coli*, LPS, 5 μg/ml, Sigma), or LPS and IL-4 (10 ng/ml, Reprotech) for four days, after which class switch recombination to IgG3 (LPS only stimulation) or IgG1 (LPS and IL-4 stimulation) was assessed using flow cytometry.

### *In vivo* cisplatin sensitivity assay

Adult, 8- to 15-week-old mice were injected intravenously with 0.8 mg/kg cisplatin or PBS. After 48 h, the bone marrow was isolated and analyzed as described below. Bone marrow from the femora was flushed out using a 21-gauge syringe with cold PBEA buffer (1 × PBS 0.5% BSA, 2 mM EDTA and 0.02% sodium azide). The samples were kept on ice. 10 × 10^6^ cells were used per staining. The following antibodies were used: Mouse Lineage Cell Detection Cocktail biotin antibody (1:40) followed by c-kit-APC (Clone 2B8, eBioscience), Streptavidin-APC-Cy7 (Southern Biotech), CD135-PE (Clone A2F10), CD48-PE-Dazzle (Clone HM48-1), 7AAD-PE-Cy5, Sca1-PE-Cy7 (Clone D7), CD34-FITC (Clone RAM34, Invitrogen, 1:100), CD127-BV421 (Clone A7R34), CD150-BV650 (Clone TC15-12F12.2), CD16/32-BV786 (clone 2.4G2, BD Bioscience). All the antibodies for FACS analysis were from Biolegend and used 1:200, unless otherwise specified. All measurements were performed with a BD LSRFortessa cell analyzer (BD Biosciences). Analyses were performed using FlowJo version 10.0.8r1.

### siRNA transfection

HCT116 and MCF-7 cells were grown in 6-well plates and transfected with SMARTpool siRNAs against human *REV1* (Horizon-Discovery) using RNAImax according to the manufacturer's specifications. Non-targeting control SMARTpool siRNAs were used as control.

### Survival assay, synergy experiments and high-throughput compound screen

For survival assays and synergy assays, cells were seeded at low confluency using the Multidrop Combi (Thermo Fisher Scientific), into 384-well plates (Greiner). After 24 h, the respective compounds were added (cisplatin, REV1i JH-RE-06, or both, or methyl methanesulfonate (MMS)) using a tecan d300e compound printer (HP), including 3–5 replicates per dosage. Positive (1 μM phenylarsine oxide) and negative (0,1% DMSO) controls were added to each assay plate. Viability readout was performed after 72 h, using the CellTiter-Blue assay (G8081/2, Promega) following the protocol of the manufacturer using an EnVision multimode plate reader (PerkinElmer). The CTB data was normalized per plate using the normalized percentage inhibition (NPI) method. NPI sets the mean of the positive control value to 0 and mean of the negative control to 1. Synergy calculations were performed in R using the Chou-Talalay Combination index method (22).

Similar protocols were used to perform the high-throughput drug screen: Using the Multidrop Combi (Thermo Fisher Scientific), untreated p53-KO lymphoma cells were seeded into 384 well plates at low confluency. After 24 h, the collection of compound libraries available at the NKI (Selleck GPCR, Kinase, Apoptosis, Phospatase, Epigenetic, LOPAC and NCI oncology) was added. This library was stored and handled as recommended by the manufacturer. Compounds from the master plate were diluted in daughter plates containing complete RPMI 1640 medium, using the microlab star liquid handling workstation (Hamilton). From the daughter plates, the diluted compounds were transferred into 384 well assay plates, in triplicate, with final concentrations of 1 μM, and 5 μM. In addition, Positive (1 μM Phenylarsine oxide) and negative (0.1% DMSO) controls were added alternately to wells in column 2 and 23 of each assay plate. After 3 days, viability was measured using CellTiter-Blue assay (G8081/2, Promega) following the protocol of the manufacturer. The CTB data was normalized per plate using the normalized percentage inhibition (NPI) method. NPI sets the mean of the positive control value to 0 and mean of the negative control to 1. Using the replicate values of both conditions a two-sided *t*-test was performed. Afterwards the *P*-values were corrected for multiple testing using the Benjamini–Hochberg method. All calculations were done in R.

### Colony formation assay

p53-KD MEFs were seeded in 10 cm dishes at various seeding densities in complete medium. One day later, the medium was removed and replaced with complete medium containing the indicated concentrations of cisplatin. After 6 days, medium was aspirated and the cells were washed with PBS and fixed in 5 ml 3:1 v/v Methanol: Acetic acid for 1 h. Following the fixation, colonies were stained by adding 3 ml of 0.3% Coomassie brilliant blue (Merck) solution prepared in H_2_O. After 1.5 h, the staining solution was removed, the dishes were washed with H_2_O and dried overnight. Colonies were counted using the Colcount (Oxford Optronix) and each condition was corrected for the plating efficiency before being normalized to the untreated condition. Data points represent the mean survival relative to the untreated control cells.

### Incucyte^®^ assays

Viable p53-knockdown (KD) MEFs were seeded at the concentration of 300 cells per well in 96-well plates. The cells were allowed to grow for 8 days or 10 days in Incucyte (Sartorius) under standard tissue culture conditions in the presence or absence of cisplatin, mitomycin C. Images were taken every 4 h and results were analyzed in Excel (Microsoft) as percentage of confluence, that represents the percentage of the image area occupied by objects.

### sgRNA design for murine *Pcna*, *Polk* and *Rev1*

Benchling was used to design guides targeting two exons or introns of each gene. These were cloned into pX330 (one guide per plasmid) or a modified form of pX330, named pX333-mCherry, which contained sites for two guides. The protocol from Ran *et al.* (23) was used to clone the guides in pX330. For pX333-mCherry, one guide was first cloned using BbsI. Plasmids were transformed into competent DH5a, sequenced using the U6 Fwd primer, and subsequently the second guide was cloned using BbsI. After transformation of DH5a with sequence-verified pX330 or pX333-mCherry, midipreps were performed using the HiPure Plasmid Midiprep kit (Invitrogen) according to the manufacturer's instructions.

### Nucleofection

To obtain WT, *Pcna^K164R^*, *Polk*-KO and *Rev1*-KO lymphoma cell lines, 10 × 10^6^ lymphoma cells were resuspended in 100 μl additive-free RPMI 1640 and resuspended with 2 times 5μg of pX330-puro each expressing a sgRNA against *Pcna*, or *Polk* and 1 μg of pmx-eGFP construct (Amaxa, Lonza), or 10 μg of pX333-mCherry featuring two guides against murine *Rev1*. Cells were nucleofected using a nucleofector II-b device (Amaxa, Lonza), using program A-30. Subsequently, cells were grown in regular medium for 2–3 days, and GFP or mCherry positive cells were sorted with MoFlo Astrios or FACSAria IIu (BD Biosciences) cell sorters and cultured for a few days. Clones were obtained via limiting dilution.

### Isolation of genomic DNA, PCR and whole exome sequencing

To obtain DNA for mouse-genotyping, and to screen for the presence of *Pcna*-mutations, *Rev1*-KO and *Polk*-KO clones, cells were lysed in lysis buffer containing ProtK overnight at 55ºC, followed by a brief inactivation of ProtK at 85ºC for 5 min. The lysate was mixed with sterile BRAUN water, of which 2 μl was used in a reaction containing MyTaq polymerase (1:100), MyTaq buffer (BioLine), and primers. DNA was amplified by PCR as indicated in Table 12.To further validate the *Polk* deletion, the genomic DNA of the clone was isolated using a genomic DNA isolation kit (Isolate II Genomic DNA kit, BioLine), followed by capture and exome sequencing using a HiSeq 2500 sequencing machine with 100 bp reads using paired end sequencing. The genome was aligned to the Mus musculus reference genome, and visualized using Integrated Genomics Viewer (IGV, version 2.8.6).

### Western blot

To validate the genetic ablation of *Rev1*, lymphoma cells were lysed in RIPA buffer (25 mM Tris–HCl (pH 7.6), 150 mM NaCl, 1% NP-40, 1% sodium deoxycholate, and 0.1% SDS) containing 1× protease inhibitor cocktail (Roche) for 30 min on ice. Lysates were sonicated for 15 min using a BioRuptor (30 s on, 30 s off, maximum power, at 4ºC). Samples were spun at 20 000 g for 10 min, and the protein concentration was measured either via a Bradford assay, or via a BCA assay. Samples were run on a 4–12% Bis–Tris gel with MES running buffer on ice, at 150–200 V for 1.5–2 h, until the loading band ran off the gel. The gels were transferred using a BioRad Transblot Turbo semi-dry transfer system with the mixed-MW program. After staining with Ponceau-S, samples were blocked for 1 h using TBS-T containing 5% skim-milk powder, followed by incubation with primary antibodies overnight at 4ºC on a roller bank in TBS-T 1% milk. The next day, blots were washed 4 times for 5 min with TBS-T, followed by a 2 h incubation with the secondary antibodies at room temperature. The membrane was washed 3 times with TBS-T for 5 min, after which the membrane was imaged on an Oddyssey scanner (LiCor). For MEFs, similar procedures were followed, but here, the subcellular fractionation kit for cultured cells (ThermoFisher scientific) was used according to the manufacturer's protocol, and the unsonicated nuclear lysate was loaded.

### *In vivo* transplantation assay

2 × 10^6^ viable p53-KO lymphoma cells were transplanted into the flank of each mouse. Briefly, cells were washed 1 time with ice-cold PBS, resuspended in 100 μl PBS, and mixed with 100 μl Cultrex reduced growth factor basement membrane extract, type 2 (R&D systems) before transplantation for the *Polk*-KO tumor transplantation experiment. Tumor growth was measured biweekly using caliper measurements, and tumor volume was calculated using the following formula: volume = 0.5 × length × width. When the tumor reached 100 mm^3^, cisplatin was injected intravenously once every two weeks (6 mg/kg) for up to 5 times. Mice were sacrificed when the tumors reached 1500 mm^3^.

### Neutral comet assay

Neutral comet assays were performed as described by Olive and Banath (24). Briefly, 8 × 10^3^ cells were diluted in 0.4 ml of PBS and were added to 1.2 ml of 1% low-gelling-temperature agarose (Sigma). Subsequently, the cell suspension was transferred onto precoated slides (Menzel-Gläser). Cell lysis was performed in neutral lysis solution (2% sarkosyl, 0.5 M Na_2_EDTA, 0.5 mg/ml proteinase K) at pH 8.0 overnight at 37°C. Slides were washed 3 times with neutral rinse and electrophoresis buffer (90 mM Tris, 90 mM boric acid, 2 mM Na2EDTA) at pH 8.5, and electrophoresis was performed in neutral rinse and electrophoresis buffer for 25 min at 20 V. Nuclei were stained with 2.5 mg/ml propidium iodide (Invitrogen) in distilled water for 20 min. Pictures of individual cells were taken with a Zeiss AxioObserver Z1 inverted microscope equipped with a cooled Hamamatsu ORCA AG black-and-white CCD camera, and were analyzed with CASP software (http://www.casplab.com). *P*-values were determined using an unpaired *t-*test with Welch's correction.

### Reconstitution of *Polk*

HEK293T cells were transfected with the pMX-GFP-mPOLK or pMX-IRES-eGFP plasmids in combination with pCL-Eco as a packaging construct, using polyethylenimine as a transfection reagent. Virus was isolated after 48 h, and frozen at -80ºC until use. To transduce the lymphoma cells, 20 μg/ml RetroNectin (Takara) was used to coat non-tissue culture plates. Cells and retrovirus were mixed 1:1 (v/v), and cells were centrifuged at 2000 rpm for 90 min at 37ºC. After passaging, cells were sorted based on GFP expression using a FACSAria Illu to obtain transduced cells.

### Statistical analysis

Statistical analyses to determine the hits from the high-throughput compound screen were done in R. All other statistical calculations were done in PRISM Graphpad (version 9.0.0).

## RESULTS

### Generation and validation of new *Rev1*-deficient mouse strains

**Figure 1.**
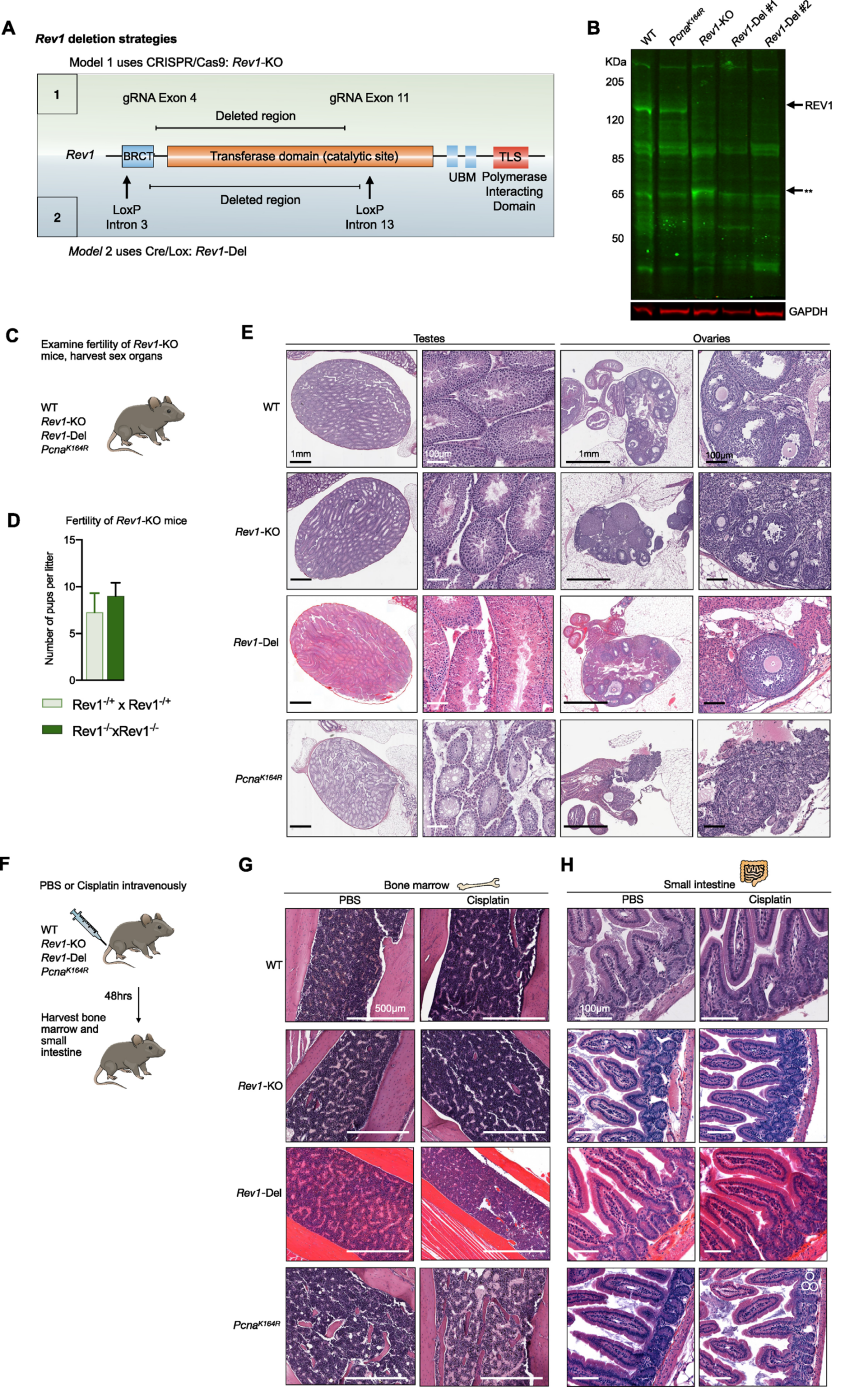
PCNA-ubiquitination but not REV1 is required for germ cell maintenance, fertility, and tolerance to cisplatin lesions. (**A**) *Rev1* was targeted using two sgRNAs that target exon 4 and exon 11 of the *Rev1* gene, truncating the BRCT-domain and Transferase domain. UBM: Ubiquitin binding motif. The deletion in *Rev1* results in the formation of an out-of-frame fusion transcript that encodes alternate amino-acid chains downstream of the fusion. Alternately, the Cre-Lox system was used to generate *Rev1*-Del mice, which have a LoxP site in intron 3 and intron 13. In this model, activation of Cre removed exon 4–13, resulting in an out-of-frame fusion transcript (see supplements). (**B**) Western blot of REV1 of WT, *Pcna^K164R^*, *Rev1*-KO and *Rev1*-Del MEFs. **Denotes a potential hypomorph REV1 protein in *Rev1*-KO MEFs. (**C**) WT, *Pcna^K164R^* and *Rev1*-KO and *Rev1*-Del mice were sacrificed, and reproductive organs were examined. To examine whether *Rev1*-KO mice featured fertility defects, heterozygous and homozygous *Rev1*-KO mice were intercrossed and litter size was examined. (**D**) Litter size of two independent heterozygous and homozygous *Rev1*-KO intercrosses reveal no fertility defects in *Rev1*-KO mice. (**E**) Pictures of ovaries and testes of WT, *Pcna^K164R^*, and *Rev1*-KO and *Rev1*-Del mice. Arrows denote to follicles. Note the absence of germ cells in *Pcna*^K164R^ cells. (**F**) WT, *Pcna^K164R^*, and *Rev1*-KO and *Rev1*-Del mice were injected intravenously with 0.8 mg/kg of cisplatin. After 48 h, the bone marrow and small intestines were isolated. (**G**) H&E staining of bone marrow of PBS and cisplatin treated mice. Note the reduction of cellularity of bone marrow cells in *Pcna^K164R^* cisplatin treated mice. Scalebars are 500 μm. (**H**) H&E staining of the small intestine of cisplatin treated mice. White circles denote apoptotic cells in the crypts of the small intestine of cisplatin treated *Pcna^K164R^* mice that were not detected in WT or *Rev1-*KO and *Rev1*-Del mice.

We have previously shown the relevance of PCNA-Ub for tolerance of cisplatin (6). *Pcna^K164R^* mice were highly sensitive to cisplatin, which ablated the hematopoietic stem and progenitor compartment (6). Because REV1 can function independent of PCNA-Ub, we wanted to examine the relevance of REV1 for ICL tolerance. To this end, we generated genetically defined *Rev1-*deficient mice. We derived a *Rev1*-KO C57BL/6 strain using CRISPR/Cas9 that featured a large region of the *Rev1* locus spanning exon 4 until exon 11, which encodes around two-thirds of the catalytic domain, and the majority of the BRCT domain (71 of 88 amino acid residues of the BRCT domain are deleted) (Figure 1A), which could be detected via PCR (Supplementary Figure S1A). The deletion resulted in frameshift mutation that introduced multiple nonsense mutations (Supplementary Figure S1B, C). Moreover, while full length REV1 protein (139KDa) was clearly detectable in MEFs from WT and *Pcna^K164R^* mutant mice it was lacking in *Rev1-*KO mice (Figure 1B). However, we did observe a protein species in *Rev1*-KO MEFs of a lower height, that was not clearly detectable in the WT MEFs. This potentially hypomorph protein migrated with a molecular weight of around 60 kDa. To exclude any residual function of a potential hypomorph REV1 in *Rev1*-KO mice, we performed functional analyses related to its catalytic activity (transferase domain) as well as the contribution of its C-terminus to immunoglobulin heavy chain class switch recombination (CSR). As REV1 substantially contributes to the formation of C/G > G/C transversions during somatic hypermutation (SHM) of immunoglobulin genes (25,26), we performed somatic hypermutation analysis *ex-vivo* on the JH4 intron region from germinal center B cells from Peyer's patches directly sorted from WT and *Rev1*-KO mice (Supplementary Figure S1D–H). In contrast to JH4 sequences from WT mice, C/G > G/C transversions were significantly reduced (Supplementary Figure S1E), in line with previous studies. Intriguingly, a strong increase was observed in G > T mutations, which we speculate is due to another SHM-associated protein that takes over for REV1 at guanines and incorporates thymidine rather than cytosines (Supplementary Figure S1E). Moreover, as *Rev1*-deficiency has been reported to reduce the efficiency of *in vitro* CSR (25,27), we examined CSR potential of WT and *Rev1*-KO naïve splenic B cells *in vitro* stimulated with lipopolysaccharide (LPS), or LPS and recombinant IL-4. Consistent with reported results, we observed a significant reduction *in vitro* CSR to IgG1 and IgG3 upon LPS, and IL-4/LPS exposure, respectively (Supplementary Figure S1F). Importantly, the CSR function of REV1 has been reported to depend on its recruitment of Ung via its C-terminal domain, suggesting that the C-terminal domain in *Rev1*-KO mice is non-functional. In addition to the *Rev1*-KO mouse, we made a *Rev1*-deficient mouse strain using the Cre-Lox system that included an even larger deletion, spanning from exon 4 until exon 13 (Figure 1A). Western blots of *Rev1*-Del MEFs did not reveal the lower hypomorph band, indicating that in these mice, REV1 is no longer produced (Figure 1B).

Taken together, our results indicate that the targeted deletion in *Rev1* locus generated two new *Rev1*-deficient strains. The large deletions prevented translation of full length REV1 in both mouse strains, and hypomorph bands were not detected in *Rev1*-Del system. Consistent, at the functional level, the absence of REV1 in *Rev1*-KO mice resulted in a reduced CSR and expected alterations in the spectrum of SHM. The sum of these data indicates that the *Rev1*-mutant mice are fully deficient for REV1.

### Germ-cells depend on PCNA-ubiquitination but not REV1

During embryogenesis starting at E12.5, but preceding meiosis, germ cell development is initiated by the migration of primordial germ cells (PGCs) to the genital ridge. During this time, PGCs are highly dependent on ICL repair, and rapidly undergo apoptosis in its absence (28). Consistent with this notion, FA patients feature reduced fertility (3), and *Fanca*-KO mice are completely infertile (28). We reasoned, that if REV1 is essential for ICL repair, germ cell maintenance and fertility of *Rev1*-KO mice should be strongly reduced. Intercrosses of both heterozygous and homozygous *Rev1*-KO mice produced viable offspring with normal litter size, arguing that *Rev1* is dispensable for germ cell development and maintenance (Figure 1C, D). We were also able to breed homozygous *Rev1*-Del mice (data not shown). In line, no germ-cell defects were observed in these *Rev1*-KO and *Rev1*-Del mice (Figure 1E). In contrast, as reported previously (14), *Pcna^K164R^* mice were completely infertile, as indicated by the absence of follicles in the ovaries and sperm and spermatogonia in the seminiferous tubules (Figure 1E).

Apparently, although REV1 can function independently of PCNA-ubiquitination, REV1 cannot suppress the deleterious effects on germ cell maintenance that arise in the absence of PCNA-K164-Ub dependent DNA damage tolerance (DDT). Furthermore, the lack of fertility-defects in *Rev1*-deficient mice indicated that REV1 is not essential for ICL repair in germ cells.

### *In vivo* tolerance to cisplatin lesions depends on PCNA-ubiquitination but not REV1

Cisplatin therapy affects highly proliferating tissues, such as the gastro-intestinal tract, and the hematopoietic system. We have previously reported that cisplatin treatment resulted in bone marrow failure and hematopoietic stem cell depletion in *Pcna^K164R^* mice (6). To examine whether *Rev1-*deficiency resulted in similar sensitivity to cisplatin *in vivo*, we administered a single dose of cisplatin (0.8 mg/kg) intravenously into WT, *Pcna^K164R^* and *Rev1*-KO and *Rev1-*Del mice, and examined the bone marrow, sternum, and gastro-intestinal tract 48 hours later by hematoxylin and eosin (H&E) staining (Figure 1F-H). Compared to WT mice, cisplatin treatment reduced the levels of nucleated cells in the bone marrow (Figure 1G) and sternum of *Pcna^K164R^* but not *Rev1*-KO and *Rev1-*Del mice (Supplementary Figure S2C), indicating that *Pcna^K164R^* nucleated cells in the bone marrow and sternum had undergone apoptosis upon cisplatin treatment. We then examined the small intestine and observed apoptotic cells in the crypts and villi of *Pcna^K164R^* but not *Rev1*-KO and *Rev1-*Del mice, which were recognizable by their pink hue, and increased nuclear condensation and fragmentation (Figure 1H). This observation in *Pcna^K164R^* but not *Rev1*-KO and *Rev1-*Del mice revealed a central role for PCNA-ubiquitination in tolerating cisplatin lesions, and that PCNA-ubiquitination and REV1-dependent DDT are likely non-epistatic.

**Figure 2.**
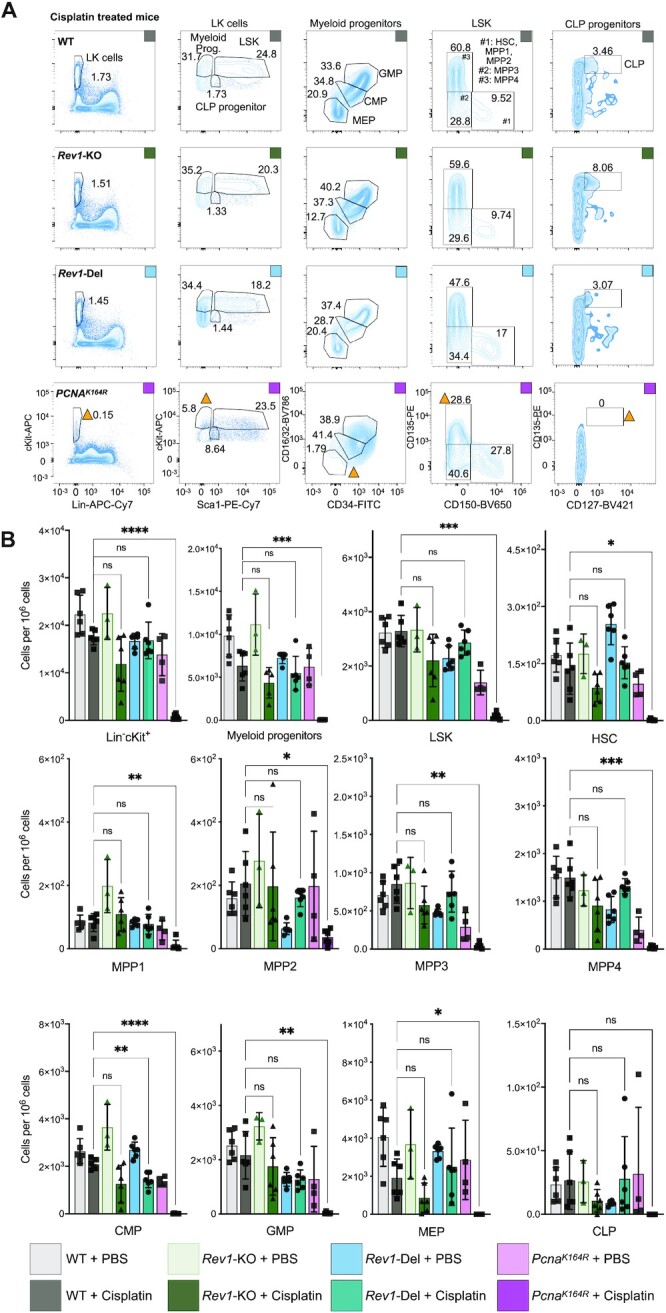
The hematopoietic system does not depend on REV1 to tolerate cisplatin lesions. (**A**) Representative gating strategy to identify hematopoietic subsets. Within the LSK compartment, an additional gating based on CD34 and CD48 was used to quantify MPP1-4 and HSCs (see **S2**). Yellow triangles indicate populations that are reduced in frequency in the *Pcna^K164R^* mice upon cisplatin treatment. (**B**) Quantification of subsets indicated by flow cytometry in WT and DDT-deficient cisplatin-treated and untreated control mice (mean ± s.d; *n* = 6, 6, 3, 6, 6, 6, 4, 6, left to right). Data were pooled from two independent experiments. *P-*values were calculated via one-way ANOVA with multiple comparisons. *P < 0.05, **P < 0.005, *** *P* < 0.0005, ****P < 0.0005.

The pancytopenia observed in severe FA patients indicates attrition of HSCs, which suggests that HSCs are highly sensitive to ICLs. Moreover, replication stress and DNA damage directly contribute to HSC demise (6,29,30). To quantitatively determine the relevance of PCNA-ubiquitination for hematopoietic stem and progenitor cells, we analyzed the composition of the bone marrow by flow cytometry after cisplatin treatment (Figure 2A; see (Supplementary Figure S2A, B for extended hematopoietic gating strategy). Compared to WT mice, cisplatin treatment significantly ablated most hematopoietic subsets analyzed (Supplementary Figure S2B), such as LK cells, LSKs, and HSCs, in *Pcna^K164R^*mice, while they were only slightly, and non-significantly reduced in *Rev1*-KO and *Rev1-*Del mice (Figure 2A, B). Moreover, the reductions in various subsets, such as HSCs, MPP1s, and MPP2s, that were detected upon cisplatin treatment were nearly identical to those observed in WT mice, indicating that *Rev1*-KO and *Rev1-*Del HSCs are not more sensitive to cisplatin.

In conclusion, the absence of a strong reduction of nucleated cells of the bone marrow and sternum, and the absence of apoptotic cells in the gastro-intestinal tract of *Rev1*-KO and *Rev1-*Del mice upon cisplatin treatment indicates that *Rev1*-deficiency does not sensitize to ICLs. REV1 is most likely not required to tolerate cisplatin-induced ICLs in the hematopoietic system, as HSCs and early progenitor subsets of *Rev1-*KO and *Rev1-*Del mice are equally sensitive as those of WT mice. While we cannot entirely exclude that the non-significant -and in case of CMPs for *Rev1-*Del mice significant- reduction of certain hematopoietic subsets in *Rev1*-KO and *Rev1-*Del mice was the result of a very minor sensitivity to cisplatin, they may also be the result of increased replication stress in hematopoietic cells that rapidly proliferate to counteract cisplatin-induced apoptosis. Furthermore, our results indicate that *in vivo*, PCNA-Ub-facilitated DDT is the predominant pathway that tolerates cisplatin lesions and that REV1 cannot compensate for a deficiency in this pathway.

### Transformed mammalian cells do not depend on REV1 for cellular fitness and tolerance to cisplatin lesions

**Figure 3.**
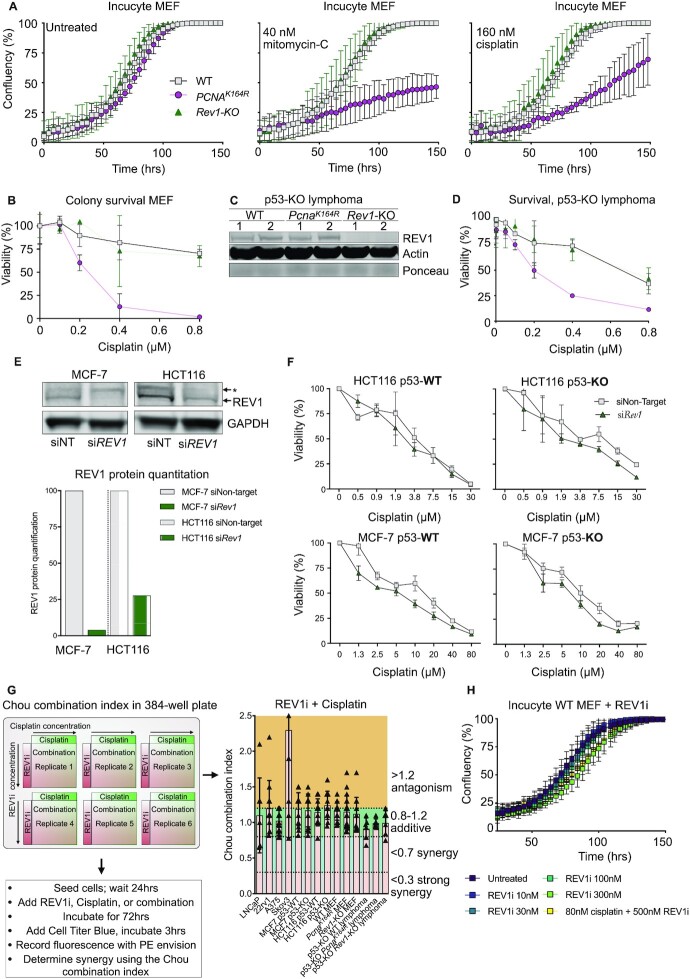
REV1 is dispensable for tolerance of cisplatin lesions in primary and transformed murine and human cells. (**A**) Proliferation of p53-KD MEFs of indicated genotypes left untreated (left panel), or incubated with 40 nM mitomycin-C (middle panel) or 160 nM cisplatin (right panel), as measured by Incucyte assay. A representative experiment of three independent experiments is shown. (**B**) Colony survival assay in p53-KD MEFs with various doses of cisplatin. A representative of three independent experiments is shown. (**C**) Western blot of REV1 in p53-KO lymphomas. (**D**) Survival assay of p53-KO lymphomas of the indicated genotype treated with cisplatin. A Representative experiment of many independent experiments is shown. (**E**) Western blot of REV1 in siRNA-mediated knockdown in HCT116 and MCF-7 cells. Protein quantification is shown on the right, levels are normalized to Actin. A representative experiment of two independent experiments is shown. Asterisk denotes non-specific band. 9. (**F**) *Rev1* was knocked down using siRNA in human HCT116 and MCF-7 colorectal and breast cancer cell lines. 24 h after transfection with the indicated siRNA, cell lines were treated for 72 hours with cisplatin after which viability was determined. A representative experiment of two independent experiments is shown. (**G**) REV1 inhibitor JH-RE-06 was co-incubated with cisplatin in the indicated human and murine cancer cell lines according to the plate layout. Synergy was determined using the Chou-Talalay -combination index. Two combined experiments are shown. (**H**) Proliferation of p53-KD WT MEFs left untreated or incubated with increasing doses of REV1i or REV1i and cisplatin, as measured by Incucyte assay.

REV1 inactivation using the small molecule inhibitor JH-RE-06 (referred from here on REV1i) has been proposed to chemosensitize cancer cells to cisplatin (12,31). To examine the relevance of REV1 during ICL repair in transformed cells *in vitro*, we determined the sensitivity of WT, *Pcna^K164R^*, and *Rev1*-KO p53-KD MEFs to interstrand crosslinking agents cisplatin and mitomycin C (MMC) by measuring confluency using Incucyte^®^ assays. Consistent with our *in vivo* data, *Pcna^K164R^* mutant but not *Rev1*-KO MEFs were hypersensitive to cisplatin and MMC (Figure 3A). Similar findings were observed using colony survival assays, again highlighting that PCNA-Ub but not REV1 is required for tolerance of cisplatin lesions (Figure 3B). To examine whether REV1 is required in tolerating cisplatin lesions in cancer cells, we genetically ablated *Rev1* in murine lymphoma cells that were described previously (18) using the same CRISPR/Cas9 targeting strategy as used for the *Rev1*-KO mice. This lymphoma model was derived from p53-KO mice, and feature a complete deletion of p53. We validated the absence of REV1 protein in *Rev1*-KO lymphomas by western blot (Figure 3C), and analyzed the sensitivity of these isogenic WT, *Pcna^K164R^* and *Rev1*-KO lymphomas to cisplatin. Consistent with our *in vivo* data and *in vitro* MEF data, *Pcna^K164R^* lymphoma cells were very sensitive to cisplatin, while *Rev1*-KO lymphomas were indistinguishable from WT (Figure 3D).

To exclude mouse to human variability with regards to REV1-sensitivity, we knocked down *Rev1* with siRNAs in human colorectal and breast cancer cell lines HCT116 and MCF-7, respectively. Analysis by western blot indicated that REV1 levels were reduced by ∼70–90% compared to cells that had been targeted using a non-targeting control (Figure 3E). We did not observe any significant difference in cisplatin sensitivity in HCT116 and MCF-7 cell lines in response to cisplatin (Figure 3F). Because p53 regulates TLS (32,33), and HCT116 and MCF-7 feature WT p53 alleles, we analyzed isogenic cell lines where p53 was ablated using CRISPR/Cas9 (19,20). Regardless of the p53 status, a *Rev1*-KD in HCT116 and MCF-7 did not enhance their sensitivity to cisplatin compared to non-targeting controls (Figure 3F).

To further exclude a function of REV1 in tolerating cisplatin-induced lesions in human cells, we performed synergy experiments using multiple doses of cisplatin and REV1i in the p53-KO murine lymphoma, human MCF-7 and HCT116, human prostate cancer cell lines 22RV1 and LNCaP, human ovarian cancer cell SKOV3, MEFs, and human melanoma cell line A375 (see Supplementary table 3A for the concentrations). We did not observe synergy between REV1i and cisplatin in any cancer cell line tested (Figure 3G), indicating that REV1 inhibition does not chemosensitize mammalian cells to ICLs. Moreover, as expected, we did not observe effects of the REV1i on WT or REV1 mutant MEFs and lymphomas (Figure 3G, H) or PreBs (data not shown).

Besides a direct role of REV1 in tolerance of genotoxic lesions, recent reports also suggested that it is required for cancer cells to prevent replication stress (34). In this context, REV1 inhibition has been reported to be toxic to cancer cells in unperturbed conditions (34). We therefore examined whether REV1 was required for cell growth. In our *Rev1*-KO p53-KD MEFs (Figure 3A) we did not observe any difference in cell growth compared to WT controls. *In vivo*, we noted a slight delay in the outgrowth of *Rev1*-KO lymphomas compared to WT controls (Supplementary Figure S3B, C). However, ultimately all mice had to be sacrificed due to tumor growth twenty days after the tumor reached 100 mm^3^ (Supplementary Figure S3C). Moreover, once established, *Rev1*-KO lymphomas did not grow slower than WT lymphomas, arguing against tumor growth defects (Supplementary Figure S3B).

To examine whether loss of p53 increased replication stress in the absence of REV1, we determined the number of DNA double strand breaks in WT and *Rev1*-KO primary and p53-KD MEFs using the neutral comet assay. Intriguingly, while in primary MEFs, *Rev1*- deficiency resulted in a significant increase of the olive tail moment, in p53-KD MEFs the tail moments between WT and *Rev1*-KO MEFs were significantly reduced (Supplementary Figure S3D), indicating that in the absence of p53, *Rev1*-deficiency does not result in increased DNA strand breaks. Importantly, the increased strand breaks in p53-proficient *Rev1-*deficient MEFs argue for a role of *Rev1* in DDT, perhaps in the tolerance of non-ICL lesions from endogenous sources.

In conclusion, REV1 is not required to tolerate cisplatin lesions in transformed murine and human cell lines. Moreover, inactivation of p53 does not result in enhanced cytotoxicity of *Rev1*-deficient cells for cisplatin. Additionally, inactivation of REV1 alone in p53-deficient conditions is not sufficient to reduce cell fitness or slow down tumor growth. Finally, in murine MEFs and mammalian tumors, PCNA-K164-ubiquitination is the predominant mode of DDT to tolerate genotoxic lesions. Our data do argue for a role of REV1 in DDT in counteracting DNA DSBs.

### The TLS polymerase Kappa promotes tolerance of cisplatin lesions *in vitro* and *in vivo*

**Figure 4.**
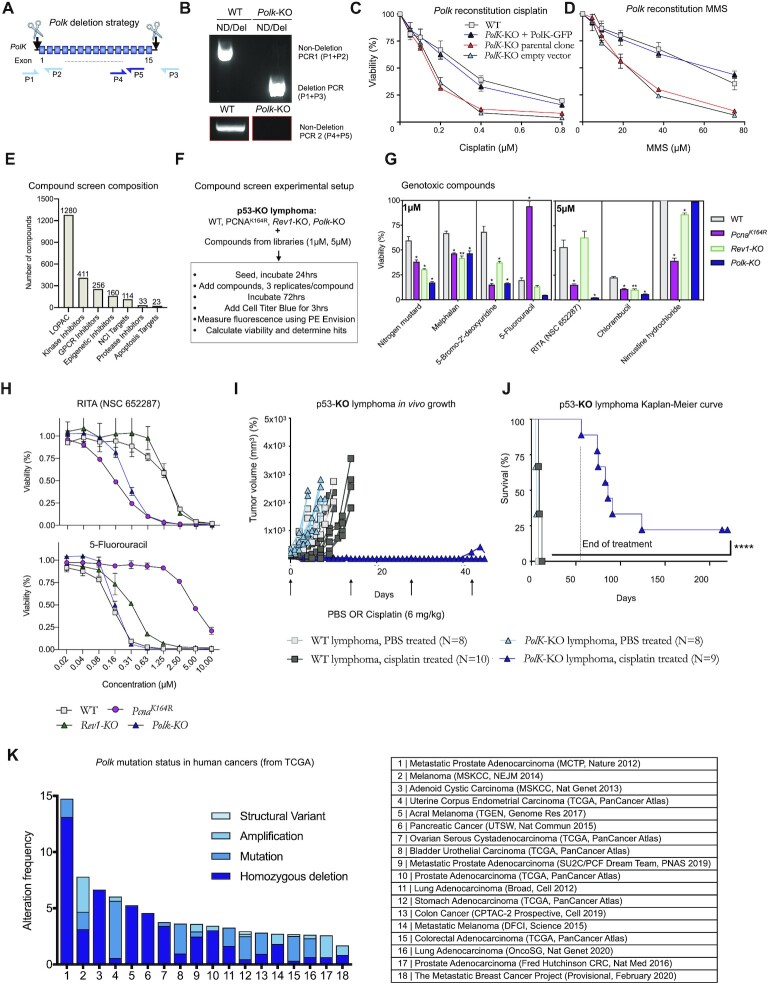
TLS polymerase kappa is frequently deleted in a subset of human cancers and tolerates DNA interstrand crosslinks. (**A**) *Polk* deletion strategy using CRISPR/Cas9 in the p53-KO lymphoma. Two guides were designed that excised the entire *Polk* gene. (**B**) PCR to validate the presence of a deleted allele (top panel) and absence of a non-deleted allele (top and bottom panels) using three primer pairs. One of two independent PCRs is shown. (C, D) *Polk*-KO clones were retrovirally transduced with *Polk* or an empty vector. Cells were treated with cisplatin (**C**) or methyl methanesulfonate (MMS) (**D**) for three days, after which the viability was determined. One representative experiment of two independent experiments is shown. (**E**) Composition of the compound screens. (**F**) Compound screen setup. (**G**) DNA damage related hits detected in the screen. Error bars denote standard deviation. *P-*values were determined via two-sided *t*-tests. The Benjammini-Hochberg method was used to correct for multiple testing. (**H**) Dose-effect response of two chosen DNA damaging agents (RITA (NSC 652287)), and 5-Fluorouracil) in the indicated cell lines. Error bars denote standard deviation. (**I**). *In vivo* tumor growth of p53-KO WT and *Polk*-KO lymphomas that have been subcutaneously transplanted. The graph starts from the moment the tumors reached 100 mm^3^, after which mice were treated once every two weeks with cisplatin (6 mg/kg) for up to five times, as indicated by arrows. (**J**) Kaplan–Meier curve of mice bearing p53-KO lymphomas of the indicated genotypes. Survival was measured from the time that tumors reached 100 mm^3^. Mice were sacrificed when the tumor reached 1500 mm^3^. *P*-values were calculated with Mantel–Cox test. **** *P* < 0.0001. (**K**) Data from the cancer genome atlas (TCGA) that depict the mutation types of *Polk* found in various human cancers.

Because REV1 was not required to tolerate cisplatin lesions, we hypothesized that other Y-family polymerases were likely recruited by PCNA-mUb to enable effective TLS of cisplatin lesions. Previous reports have shown POLK to be required to tolerate cisplatin lesions in *Xenopus* egg extracts and human cells, but results in murine cells (35–39) were not consistent. We therefore investigated POLK as a candidate and deleted both alleles of *Polk* by CRISPR/Cas9 in our isogenic murine DDT lymphoma model (Figure 4A). The *Polk*-KO was validated by genomic PCR-based analyses and subsequently validated by whole exome sequencing (Figure 4B, Supplementary Figure S4A–C).

Having established an isogenic *Polk*-KO lymphoma subclone, we compared its cisplatin sensitivity to the WT parental clone. Importantly, we detected a striking increase in the sensitivity of *Polk-*deficient lymphoma cells to both cisplatin and the alkylating agent MMS (Figure 4C, D). Retroviral reconstitution of *Polk* desensitized the *Polk*-KO lymphoma, indicating that its hypersensitivity was solely dictated by the lack of POLK (Figure 4C, D). Moreover, requirement for POLK to tolerate genotoxic lesions was further demonstrated by unbiased high-throughput compound screens (Figure 4E–G, Supplementary Figure S4D–F), where *Polk*-KO cells were found to be highly sensitive to the ICL-inducing agent RITA (NSC 652287), the alkylating agent nitrogen mustard, and the toxic nucleotide analog Bromodeoxyuridine (BrdU) (Figure 4E–G), to which *Rev1*-KO cells were sensitive, although to a less extent than *Pcna^K164R^* cells. The sensitivity of the cell lines to several identified compounds was subsequently validated by performing a dose-response curve. The dose-response curve frequently matched the sensitivity observed in the initial screen, further validating the sensitivity of the initial compound screen (Figure 4H). However, in the dose response curve of RITA (NSC 652287), we did not detect enhanced sensitivity of *Rev1-*KO cells, arguing against the relevance of REV1 in tolerating RITA-induced lesions (Figure 4H). Intriguingly, we picked up and validated a strong insensitivity of *Pcna^K164R^* cells, though not *PolK-*KO cells, to 5-Fluorouracil (5-FU), indicating that while POLK is required to tolerate ICLs, toxicity of 5-FU is caused by PCNA-ubiquitination independent of POLK (Figure 4H). Intriguingly, we identified *Pcna^K164R^*, *Rev1*-KO and *Polk*-KO clones to be sensitive to various types of steroid hormones, such as 17a-hydroxytamoxifen and progesterone (Supplementary Figure S4G–I), which are known to induce DNA adducts that DDT-deficient DT40 cells are sensitive to (40). The steroid-hormone related hits suggest that specific TLS polymerases are required to tolerate specific adducts created by steroid hormones, and finally suggests the specific type of enigmatic endogenously produced lesions that REV1 and POLK are required to tolerate. Importantly, the data indicate the high relevance of POLK to tolerate DNA damage induced by many classical chemotherapeutic agents and suggests that POLK is a key TLS polymerase that tolerates a wide range of genotoxic lesions.

Because *Polk*-KO cells were highly sensitive to ICL-inducing agents, we tested *Polk-*deficiency as a cancer specific vulnerability to cisplatin treatment. We subcutaneously transplanted isogenic WT and *Polk*-deficient lymphomas in nude mice to exclude any immune-mediated rejections. Tumor growth was followed using caliper measurements. Regarding vehicle controls, WT and *Polk*-KO lymphoma grew out rapidly and established large tumors within seven days. Consequently, all mice of the vehicle control groups had to be sacrificed within seven days after the start of treatment, after the tumors had reached a maximum allowed size of 1500 mm^3^ (Figure 4I, J).

Notably, while the WT lymphomas could not be controlled by a clinical dose of cisplatin, eight out of nine mice that had received *Polk*-deficient lymphomas survived five cycles of cisplatin treatment, and the tumor size reduced to nearly undetectable levels during the treatment phase (Figure 4I, J). In seven out of nine *Polk*-KO tumor bearing mice, the tumors grew out within fifty days after cessation of treatment. In two out of nine mice however, tumors remained undetectable for up to two hundred days after the start of treatment. As homozygous inactivating deletions of *Polk* are relatively frequently observed in human cancers, such as prostate, ovarian, and pancreatic cancers as well as melanoma (Figure 4K), our data suggest that these patients may benefit from treatments with platinating agents.

In conclusion, the genetic knockout of *Polk* in a p53-KO murine lymphoma strongly enhanced the cytotoxicity of cisplatin, resulting in marked increased in the overall survival time of *Polk*-KO tumor bearing mice that were treated with cisplatin. This preclinical mouse model reveals a unique opportunity for precision cancer medicine in predicting cisplatin responsiveness based on the *Polk* status.

## DISCUSSION

Knowledge on ICL repair has expanded greatly in the past few years. The direct role of TLS in FA-mediated ICL repair highlights DDT as a critical arm of the DNA damage response network. Inhibition of DDT is being considered to enhance the treatment efficacy of platinating agents to treat cancer. These findings implicated that targeting of specific DDT components provides an attractive mode to chemosensitize tumors to platinating agents (12,13,18,31,34,41).

PCNA-mUb and REV1 are considered as essential to recruit TLS polymerases (8–10,42). However, the division of labor between PCNA-Ub-dependent DDT and REV1-dependent DDT in mammalian cells has been unclear. Here, we directly compared the relevance of PCNA-ubiquitination-dependent and REV1-dependent DDT in mice as well as human and mouse cell lines. Our studies in mice and in mammalian cells strongly indicate PCNA-ubiquitination is the predominant mode of DDT, and that a genetic ablation of REV1 does not sensitize murine and human cancer cells to various classes of clinically approved DNA damaging agents. Along these lines, our analyses in the mammalian system exclude REV1 as being critical in coordinating replicative bypass of cisplatin-induced DNA lesions. Instead, our data identify the TLS polymerase POLK as an important DDT component that tolerates cisplatin lesions. Lack of POLK but not REV1 rendered human and mouse cell lines hypersensitive to cisplatin treatment. Loss of *Polk* strongly amplified the sensitivity to a cisplatin-monotherapy in an isogenic lymphoma transplantation model, resulting in robust longitudinal control of tumor outgrowth in all mice, as well as a total tumor clearance in two out of nine mice. Stable reconstitution of *Polk* rescued cisplatin sensitivity of *Polk*-KO lymphomas. According to the TCGA database, homozygous inactivation mutations in the *Polk* gene are relatively frequent in human prostate cancer, melanoma, and endometrial cancers. This notion in combination with our findings implies that the *Polk* status provides a novel parameter in predicting a tumor-specific hyper-responsiveness to cisplatin and screening of the *Polk* status in cancers likely provides an attractive avenue for personalized cancer medicine.

We previously reported that PCNA-ubiquitination is essential for the tolerance of ICLs, *in vivo* and *in vitro* (6,43). Indeed, *Pcna^K164R^* mice were hypersensitive to cisplatin, as indicated by a strong and significant reduction of nucleated cells in the bone marrow and sternum and the presence of elevated numbers of apoptotic cells in the gut. Moreover, murine primary, immortalized, and transformed cells that carried the *Pcna^K164R^* mutation were hypersensitive to various genotoxic compounds *in vitro*, such as cisplatin and MMC. These data agree with previous reports in human and avian cell lines (9,44), indicating a central role of PCNA-Ub dependent DDT in higher organisms.

In contrast, this direct comparative study between *Pcna^K164R^* and *Rev1-*deficient systems revealed that REV1 is dispensable for the tolerance of ICLs in the mammalian system. Our results are somewhat puzzling given the many reports indicating the relevance of REV1 in ICL repair (not extensive: 12,31,34,45,46). However, we provide several lines of evidence *in vivo*, *ex vivo* and *in vitro* to corroborate this important conclusion. First, in our defined *Rev1-*KO mouse model, we were able to recapitulate the SHM nucleotide substitution pattern and reduced level of CSR that were established prior in independent *Rev1-*deficient mouse models (25–27). Second, we did not observe significant decreases of various hematopoietic subsets of cells in the bone marrow of *Rev1*-deficient mice as compared to WT mice after cisplatin treatment, while *Pcna^K164R^* mice had strong reductions of all subsets measured. Third, we noted that *Pcna^K164R^* mice, but not *Rev1-*mutant mice were infertile. Germ cells critically depend on ICL repair, as indicated by the absence or reduction of germ cells when ICL repair is impaired (28), and the high sensitivity of germ cell-derived tumors to cisplatin (47). Our findings further demonstrate that cells most sensitive to ICLs, *i.e*. stem cells, are not affected by loss of *Rev1*. The use of genetically defined mouse models and independent cell lines thereof exclude confounding effects that may result from genetic drifts. Fourth, the absence of sensitivity to ICLs *in vitro* of multiple *Rev1-*deficient murine and human transformed, and non-transformed cell lines argue against any supposed sensitivity being masked due to a difference in species, genetic makeup of the cell line, or whether cells are transformed or not. Finally, the multiple manners in which REV1 function was attenuated, either by CRISPR/Cas9-mediated KO, siRNA-mediated KD, or via direct inhibition using small molecule inhibitor JH-RE-06, excludes that effects are masked by isolation of a particular resistant clone, and further indicated that REV1 is not essential for tolerating ICLs. Of note, while cisplatin and MMC are potent inducers of interstrand crosslinks, the majority of lesions introduced are in fact the much less toxic intrastrand crosslinks and adducts. While we cannot clearly distinguish the effects of *inter*strand and *intra*strand crosslinks in our assays, our *in vivo* and *in vitro* results clearly demonstrate that REV1 is not important to tolerate ICLs.

Our data indicate that inhibition of DDT to treat cancer would carry the risk of introducing potentially severe adverse effects, such as bone marrow failure, or enhanced levels of DNA damage (6). However, our data also imply that tumor- specific DDT defects provide unique opportunities for precision cancer medicine. Indeed, we observed a strong enhancement of cisplatin cytotoxicity in *Polk*-deficient lymphoma cells, which could be rescued by retroviral transduction of *Polk*. Our data agree with findings of Gautier *et al.*, who demonstrated that POLK was essential for replication through unhooked ICLs (38). Interestingly, POLK appears to function in every phase of the cell cycle (38,41,48,49), increasing the range and efficacy of potential treatments targeting *Polk*-deficient tumors. Especially, the G1 activity would allow for the targeting of non-cycling dormant cancer stem cells, which would otherwise be protected from the cytotoxic effects of cisplatin and which could reoccur after cessation of treatment (50). In addition, as *Polk*-deficient cancer cells are sensitive to different classes of DNA damaging agents, recurrence due to acquired resistance of such cancer cells to one class of cytotoxic drugs could be mitigated by switching to a different class of DNA damaging agents. Importantly, we established the *Polk*-KO clone via limiting dilution, a process which takes roughly two months. During this time, and during culturing, *Polk*-KO cells did not become resistant to cisplatin, or developed other resistance mechanisms, which probably contributed to the extensive sensitivity of *Polk*-KO lymphomas to cisplatin *in vivo*. Intriguingly, because cisplatin introduces a plethora of different lesions in the DNA, perhaps the high sensitivity of *Polk*-deficient cells to cisplatin can also be explained by the requirement for POLK to tolerate a wide range of genotoxic lesions. For metastatic prostate cancer, current standard of treatment consists of a combination of androgen-receptor inhibitors and taxanes (51), which leaves platinating and alkylating agents open as an alternative treatment option, or as an adjuvant therapy.

Inhibition of DNA damage tolerance pathways is currently under intense investigation. Indeed, preclinical data shows promising results for the inhibition of RAD6B (13), or the RPA ubiquitination enzyme RFWD3 (52), both of which enhance the cytotoxicity of cisplatin. Other TLS polymerases besides POLK that could be required for ICL repair could be POLH, which is able to extend past ICLs (45,53,54) and whose knockout sensitizes cells to cisplatin, and REV3/REV7 (45,55–57), as *Rev3-*KO cells are hypersensitive to DNA damage. These results also indicate the non-redundant requirement of multiple TLS polymerases and DDT pathways to tolerate cisplatin lesions. In line with this observation, we observed that *Pcna^K164R^* cells were more sensitive than *Polk*-deficient cells for cisplatin, arguing that distinct DDT-pathways or TLS polymerases act on cisplatin lesions. Perhaps the differential requirement of TLS polymerases to tolerate cisplatin lesions depend on the genomic context, cell cycle, or alternatively, distinct polymerases operate sequentially.

Platinating and alkylating agents are mainstays in current cancer treatments, and their synergistic action in combination with immune checkpoint inhibitors has resulted in renewed interest in the use of these compounds for the treatment of solid and hematological malignancies (58). Our data indicate that cancer-specific defects in the DDT system represent clinically actionable targets that can be exploited for personalized cancer medicine. In conclusion, we show that PCNA-ubiquitination, but not REV1, is required for tolerance of cisplatin induced ICLs in mammalian cells. Future studies should examine whether the loss of *Polk* in human cancers can be exploited in human cancer therapy and predict responsiveness to platinating agents, and whether cisplatin sensitivity of *Polk-*deficient cells is caused by sensitivity to cisplatin-induced intrastrand or interstrand crosslinks.

## DATA AVAILABILITY

Flow Cytometry data has been deposited on the FlowRepository website (see https://flowrepository.org/id/FR-FCM-Z5K6). Whole genome sequencing data of a WT p53-KO lymphoma clone, and whole exome sequencing data of the *PolK*-KO clone can be found at: https://dataview.ncbi.nlm.nih.gov/object/PRJNA854140?reviewer=k50bkvh4lnqn09n1a5hp4pl2g1, using accession numbers SRR19901279 (for the WT lymphoma clone), and SRR19901280 (for the *Polk*-KO clone).

## Supplementary Material

gkac545_Supplemental_Files

## References

[1] Deans A.J. , West S.C. DNA interstrand crosslink repair and cancer. Nat. Rev. Cancer. 2011; 11:467–480. 21701511 10.1038/nrc3088 PMC3560328

[2] Ceccaldi R. , Sarangi P., D’Andrea A.D The fanconi anaemia pathway: new players and new functions. Nat. Rev. Mol. Cell Biol. 2016; 17:337–349. 27145721 10.1038/nrm.2016.48

[3] Tsui V. , Crismani W. The fanconi anemia pathway and fertility. Trends Genet. 2019; 35:199–214. 30683429 10.1016/j.tig.2018.12.007

[4] Kottemann M.C. , Smogorzewska A. Fanconi anaemia and the repair of watson and crick DNA crosslinks. Nature. 2013; 493:356–363. 23325218 10.1038/nature11863 PMC3700363

[5] Räschle M. , Knipscheer P., Knipsheer P., Enoiu M., Angelov T., Sun J., Griffith J.D., Ellenberger T.E., Schärer O.D., Walter J.C. Mechanism of replication-coupled DNA interstrand crosslink repair. Cell. 2008; 134:969–980. 18805090 10.1016/j.cell.2008.08.030 PMC2748255

[6] Pilzecker B. , Buoninfante O.A., van den Berk P., Lancini C., Song J.Y., Citterio E., Jacobs H. DNA damage tolerance in hematopoietic stem and progenitor cells in mice. Proc. Natl. Acad. Sci. U.S.A. 2017; 114:E6875–E6883. 28761001 10.1073/pnas.1706508114 PMC5565453

[7] Hoege C. , Pfander B., Moldovan G.L., Pyrowolakis G., Jentsch S. RAD6-dependent DNA repair is linked to modification of PCNA by ubiquitin and SUMO. Nature. 2002; 419:135–141. 12226657 10.1038/nature00991

[8] Mailand N. , Gibbs-Seymour I., Bekker-Jensen S. Regulation of PCNA-protein interactions for genome stability. Nat. Rev. Mol. Cell Biol. 2013; 14:269–282. 23594953 10.1038/nrm3562

[9] Edmunds C.E. , Simpson L.J., Sale J.E. PCNA ubiquitination and REV1 define temporally distinct mechanisms for controlling translesion synthesis in the avian cell line DT40. Mol. Cell. 2008; 30:519–529. 18498753 10.1016/j.molcel.2008.03.024

[10] Ross A.L. , Simpson L.J., Sale J.E. Vertebrate DNA damage tolerance requires the C-terminus but not BRCT or transferase domains of REV1. Nucleic Acids Res. 2005; 33:1280–1289. 15741181 10.1093/nar/gki279 PMC552965

[11] Martín-Pardillos A. , Tsaalbi-Shtylik A., Chen S., Lazare S., van Os R.P., Dethmers-Ausema A., Fakouri N.B., Bosshard M., Aprigliano R., van Loon B. et al. Genomic and functional integrity of the hematopoietic system requires tolerance of oxidative DNA lesions. Blood. 2017; 130:1523–1534. 28827409 10.1182/blood-2017-01-764274 PMC5620415

[12] Wojtaszek J.L. , Chatterjee N., Najeeb J., Ramos A., Lee M., Bian K., Xue J.Y., Fenton B.A., Park H., Li D. et al. A small molecule targeting mutagenic translesion synthesis improves chemotherapy. Cell. 2019; 178:152–159. 31178121 10.1016/j.cell.2019.05.028 PMC6644000

[13] Sanders M.A. , Haynes B., Nangia-Makker P., Polin L.A., Shekhar M.P. Pharmacological targeting of RAD6 enzyme-mediated translesion synthesis overcomes resistance to platinum-based drugs. J. Biol. Chem. 2017; 292:10347–10363. 28490629 10.1074/jbc.M117.792192 PMC5481549

[14] Langerak P. , Nygren A.O.H., Krijger P.H.L., van den Berk P.C.M., Jacobs H. A/T mutagenesis in hypermutated immunoglobulin genes strongly depends on PCNAK164 modification. J. Exp. Med. 2007; 204:1989–1998. 17664295 10.1084/jem.20070902 PMC2118671

[15] Rolink A. , Kudo A., Karasuyama H., Kikuchi Y., Melchers F. Long-term proliferating early pre b cell lines and clones with the potential to develop to surface Ig-positive, mitogen reactive b cells in vitro and in vivo. EMBO J. 1991; 10:327–336. 1991449 10.1002/j.1460-2075.1991.tb07953.x PMC452650

[16] Abbondanzo S.J. , Gadi I., Stewart C.L. 49]Derivation of embryonic stem cell lines. Guide to Techniques in Mouse Development, Methods in Enzymology. 1993; 225:Academic Press 803–823. 10.1016/0076-6879(93)25052-4 8231888

[17] Dirac A.M.G. , Bernards R. Reversal of senescence in mouse fibroblasts through lentiviral suppression of p53. J. Biol. Chem. 2003; 278:11731–11734. 12551891 10.1074/jbc.C300023200

[18] Buoninfante O.A. , Pilzecker B., Aslam M.A., Zavrakidis I., van der Wiel R., van de Ven M., van den Berk P.C.M., Jacobs H. Precision cancer therapy: profiting from tumor specific defects in the DNA damage tolerance system. Oncotarget. 2018; 9:18832–18843. 29721165 10.18632/oncotarget.24777 PMC5922359

[19] Wellenstein M.D. , Coffelt S.B., Duits D.E.M., van Miltenburg M.H., Slagter M., de Rink I., Henneman L., Kas S.M., Prekovic S., Hau C.S. et al. Loss of p53 triggers WNT-dependent systemic inflammation to drive breast cancer metastasis. Nature. 2019; 572:538–542. 31367040 10.1038/s41586-019-1450-6 PMC6707815

[20] Wang C. , Vegna S., Jin H., Benedict B., Lieftink C., Ramirez C., de Oliveira R.L., Morris B., Gadiot J., Wang W. et al. Inducing and exploiting vulnerabilities for the treatment of liver cancer. Nature. 2019; 574:268–272. 31578521 10.1038/s41586-019-1607-3 PMC6858884

[21] Jolly C.J. , Klix N., Neuberger M.S. Rapid methods for the analysis of immunoglobulin gene hypermutation: application to transgenic and gene targeted mice. Nucleic Acids Res. 1997; 25:1913–1919. 9115357 10.1093/nar/25.10.1913 PMC146691

[22] Chou T. Drug combination studies and their synergy quantification using the chou-talalay method. Cancer Res. 2010; 70:440–447. 20068163 10.1158/0008-5472.CAN-09-1947

[23] Ran F.A. , Hsu P.D., Wright J., Agarwala V., Scott D.A., Zhang F. Genome engineering using the CRISPR-Cas9 system. Nat. Protoc. 2013; 8:2281–2308. 24157548 10.1038/nprot.2013.143 PMC3969860

[24] Olive P.L. , Banáth J.P. The comet assay: a method to measure DNA damage in individual cells. Nat. Protoc. 2006; 1:23–29. 17406208 10.1038/nprot.2006.5

[25] Krijger P.H. , Tsaalbi-Shtylik A., Wit N., van den Berk P.C.M., de Wind N., Jacobs H. Rev1 is essential in generating g to c transversions downstream of the ung2 pathway but not the msh2+ung2 hybrid pathway. Eur. J. Immunol. 2013; 43:2765–2770. 23857323 10.1002/eji.201243191

[26] Jansen J.G. , Langerak P., Tsaalbi-Shtylik A., van den Berk P., Jacobs H., de Wind N. Strand-biased defect in C/G transversions in hypermutating immunoglobulin genes in Rev1-deficient mice. J. Exp. Med. 2006; 203:319–323. 16476771 10.1084/jem.20052227 PMC2118202

[27] Zan H. , White C.A., Thomas L.M., Mai T., Li G., Xu Z., Zhang J., Casali P. Rev1 recruits ung to switch regions and enhances dU glycosylation for immunoglobulin class switch DNA recombination. Cell Rep. 2012; 2:1220–1232. 23140944 10.1016/j.celrep.2012.09.029 PMC3518390

[28] Hill R.J. , Crossan G.P. DNA cross-link repair safeguards genomic stability during premeiotic germ cell development. Nat. Genet. 2019; 51:1283–1294. 31367016 10.1038/s41588-019-0471-2 PMC6675612

[29] Flach J. , Bakker S.T., Mohrin M., Conroy P.C., Pietras E.M., Reynaud D., Alvarez S., Diolaiti M.E., Ugarte F., Forsberg E.C. et al. Replication stress is a potent driver of functional decline in ageing haematopoietic stem cells. Nature. 2014; 512:198–202. 25079315 10.1038/nature13619 PMC4456040

[30] Walter D. , Lier A., Geiselhart A., Thalheimer F.B., Huntscha S., Sobotta M.C., Moehrle B., Brocks D., Bayindir I., Kaschutnig P. et al. Exit from dormancy provokes DNA-damage-induced attrition in haematopoietic stem cells. Nature. 2015; 520:549–552. 25707806 10.1038/nature14131

[31] Chatterjee N. , Whitman M.A., Harris C.A., Min S.M., Jonas O., Lien E.C., Luengo A., Vander Heiden M.G., Hong J., Zhou P. et al. REV1 inhibitor JH-RE-06 enhances tumor cell response to chemotherapy by triggering senescence hallmarks. Proc. Natl. Acad. Sci. U.S.A. 2020; 117:28918–28921. 33168727 10.1073/pnas.2016064117 PMC7682577

[32] Avkin S. , Sevilya Z., Toube L., Geacintov N., Chaney S.G., Oren M., Livneh Z. p53 and p21 regulate error-prone DNA repair to yield a lower mutation load. Mol. Cell. 2006; 22:407–413. 16678112 10.1016/j.molcel.2006.03.022

[33] Hampp S. , Kiessling T., Buechle K., Mansilla S.F., Thomale J., Rall M., Ahn J., Pospiech H., Gottifredi V., Wiesmüller L. DNA damage tolerance pathway involving DNA polymerase ι and the tumor suppressor p53 regulates DNA replication fork progression. Proc. Natl. Acad. Sci. U.S.A. 2016; 113:E4311–E4319. 27407148 10.1073/pnas.1605828113 PMC4968756

[34] Nayak S. , Calvo J.A., Cong K., Peng M., Berthiaume E., Jackson J., Zaino A.M., Vindigni A., Hadden M.K., Cantor S.B. Inhibition of the translesion synthesis polymerase REV1 exploits replication gaps as a cancer vulnerability. Sci. Adv. 2020; 6:eaaz7808. 32577513 10.1126/sciadv.aaz7808 PMC7286678

[35] Kanemaru Y. , Suzuki T., Sassa A., Matsumoto K., Adachi N., Honma M., Numazawa S., Nohmi T. DNA polymerase kappa protects human cells against MMC-induced genotoxicity through error-free translesion DNA synthesis. Genes Environ. 2017; 39:6. 28077981 10.1186/s41021-016-0067-3 PMC5219776

[36] Zhuo M. , Gorgun M.F., Englander E.W. Translesion synthesis DNA polymerase kappa is indispensable for DNA repair synthesis in cisplatin exposed dorsal root ganglion neurons. Mol. Neurobiol. 2018; 55:2506–2515. 28391554 10.1007/s12035-017-0507-5 PMC5632588

[37] Jha V. , Ling H. Structural basis for human DNA polymerase kappa to bypass cisplatin intrastrand cross-link (Pt-GG) lesion as an efficient and accurate extender. J. Mol. Biol. 2018; 430:1577–1589. 29715472 10.1016/j.jmb.2018.04.023

[38] Williams H.L. , Gottesman M.E., Gautier J. Replication-Independent repair of DNA interstrand crosslinks. Mol. Cell. 2012; 47:140–147. 22658724 10.1016/j.molcel.2012.05.001 PMC3666337

[39] Wit N. , Buoninfante O.A., Van Den Berk P.C.M., Jansen J.G., Hogenbirk M.A., De Wind N., Jacobs H Roles of PCNA ubiquitination and TLS polymerases κ and η in the bypass of methyl methanesulfonate-induced DNA damage. Nucleic Acids Res. 2015; 43:282–294. 25505145 10.1093/nar/gku1301 PMC4288191

[40] Mizutani A. , Okada T., Shibutani S., Sonoda E., Hochegger H., Nishigori C., Miyachi Y., Takeda S., Yamazoe M. Extensive chromosomal breaks are induced by tamoxifen and estrogen in DNA repair-deficient cells. Cancer Res. 2004; 64:3144–3147. 15126352 10.1158/0008-5472.can-03-3489

[41] Yang Y. , Gao Y., Mutter-Rottmayer L., Zlatanou A., Durando M., Ding W., Wyatt D., Ramsden D., Tanoue Y., Tateishi S. et al. DNA repair factor RAD18 and DNA polymerase Polκ confer tolerance of oncogenic DNA replication stress. J. Cell Biol. 2017; 216:3097–3115. 28835467 10.1083/jcb.201702006 PMC5626543

[42] Guo C. , Fischhaber P.L., Luk-Paszyc M.J., Masuda Y., Zhou J., Kamiya K., Kisker C., Friedberg E.C. Mouse rev1 protein interacts with multiple DNA polymerases involved in translesion DNA synthesis. EMBO J. 2003; 22:6621–6630. 14657033 10.1093/emboj/cdg626 PMC291821

[43] Krijger P.H.L. , Van Den Berk P.C.M., Wit N., Langerak P., Jansen J.G., Reynaud C.A., De Wind N., Jacobs H PCNA ubiquitination-independent activation of polymerase η during somatic hypermutation and DNA damage tolerance. DNA Repair (Amst.). 2011; 10:1051–1059. 21889916 10.1016/j.dnarep.2011.08.005

[44] Thakar T. , Leung W., Nicolae C.M., Clements K.E., Shen B., Bielinsky A.K., Moldovan G.L. Ubiquitinated-PCNA protects replication forks from DNA2-mediated degradation by regulating okazaki fragment maturation and chromatin assembly. Nat. Commun. 2020; 11:2147. 32358495 10.1038/s41467-020-16096-w PMC7195461

[45] Hicks J.K. , Chute C.L., Paulsen M.T., Ragland R.L., Howlett N.G., Guéranger Q., Glover T.W., Canman C.E. Differential roles for DNA polymerases eta, zeta, and REV1 in lesion bypass of intrastrand versus interstrand DNA cross-links. Mol. Cell. Biol. 2010; 30:1217–1230. 20028736 10.1128/MCB.00993-09 PMC2820889

[46] Xie K. , Doles J., Hemann M.T., Walker G.C. Error-prone translesion synthesis mediates acquired chemoresistance. Proc. Natl. Acad. Sci. U.S.A. 2010; 107:20792–20797. 21068378 10.1073/pnas.1011412107 PMC2996453

[47] Richardson L.C. , Neri A.J., Tai E., Glenn J.D. Testicular cancer: a narrative review of the role of socioeconomic position from risk to survivorship. Urol. Oncol. 2012; 30:95–101. 22127018 10.1016/j.urolonc.2011.09.010 PMC4698969

[48] Ogi T. , Limsirichaikul S., Overmeer R.M., Volker M., Takenaka K., Cloney R., Nakazawa Y., Niimi A., Miki Y., Jaspers N.G. et al. Three DNA polymerases, recruited by different mechanisms, carry out NER repair synthesis in human cells. Mol. Cell. 2010; 37:714–727. 20227374 10.1016/j.molcel.2010.02.009

[49] Xiang Y. , Laurent B., Hsu C.-H., Nachtergaele S., Lu Z., Sheng W., Xu C., Chen H., Ouyang J., Wang S. et al. RNA m6A methylation regulates the ultraviolet-induced DNA damage response. Nature. 2017; 543:573–576. 28297716 10.1038/nature21671 PMC5490984

[50] Batlle E. , Clevers H. Cancer stem cells revisited. Nat. Med. 2017; 23:1124–1134. 28985214 10.1038/nm.4409

[51] Linder S. , Van Der Poel H.G., Bergman A.M., Zwart W., Prekovic S. Enzalutamide therapy for advanced prostate cancer: efficacy, resistance and beyond. Endocr. Relat. Cancer. 2019; 26:R31–R52. 10.1530/ERC-18-0289 PMC6215909 30382692

[52] Gallina I. , Hendriks I.A., Hoffmann S., Larsen N.B., Johansen J., Colding-Christensen C.S., Schubert L., Sellés-Baiget S., Fábián Z., Kühbacher U. et al. The ubiquitin ligase RFWD3 is required for translesion DNA synthesis. Mol. Cell. 2021; 81:442–458. 33321094 10.1016/j.molcel.2020.11.029 PMC7864614

[53] Silverstein T.D. , Johnson R.E., Jain R., Prakash L., Prakash S., Aggarwal A.K. Structural basis for the suppression of skin cancers by DNA polymerase eta. Nature. 2010; 465:1039–1043. 20577207 10.1038/nature09104 PMC3030469

[54] Bassett E. , King N.M., Bryant M.F., Hector S., Pendyala L., Chaney S.G., Cordeiro-Stone M. The role of DNA polymerase η in translesion synthesis past platinum-DNA adducts in human fibroblasts. Cancer Res. 2004; 64:6469–6475. 15374956 10.1158/0008-5472.CAN-04-1328

[55] Huang K.K. , Jang K.W., Kim S., Kim H.S., Kim S.-M., Kwon H.J., Kim H.R., Yun H.J., Ahn M.J., Park K.U. et al. Exome sequencing reveals recurrent REV3L mutations in cisplatin-resistant squamous cell carcinoma of head and neck. Sci. Rep. 2016; 6:19552. 26790612 10.1038/srep19552 PMC4726344

[56] Yang L. , Shi T., Liu F., Ren C., Wang Z., Li Y., Tu X., Yang G., Cheng X. REV3L, a promising target in regulating the chemosensitivity of cervical cancer cells. PLoS One. 2015; 10:e0120334. 25781640 10.1371/journal.pone.0120334 PMC4364373

[57] Doles J. , Oliver T.G., Cameron E.R., Hsu G., Jacks T., Walker G.C., Hemann M.T. Suppression of Rev3, the catalytic subunit of Pol{zeta}, sensitizes drug-resistant lung tumors to chemotherapy. Proc. Natl. Acad. Sci. U.S.A. 2010; 107:20786–20791. 21068376 10.1073/pnas.1011409107 PMC2996428

[58] Voorwerk L. , Slagter M., Horlings H.M., Sikorska K., van de Vijver K.K., de Maaker M., Nederlof I., Kluin R.J.C., Warren S., Ong S.F. et al. Immune induction strategies in metastatic triple-negative breast cancer to enhance the sensitivity to PD-1 blockade: the TONIC trial. Nat. Med. 2019; 25:920–928. 31086347 10.1038/s41591-019-0432-4

