# Supplementary material for: Division of labor within the DNA damage tolerance system reveals non-epistatic and clinically actionable targets for precision cancer medicine
Source: Nucleic Acids Res. 2022 Jul 12;50(13):7420–35. doi: 10.1093/nar/gkac545 (PMC9303390; doi:10.1093/nar/gkac545)
Supplement: gkac545_Supplemental_Files [file gkac545_supplemental_files.zip › Rev1 supplements for NAR-2.pdf]

Supplementary figure legends:

Figure S1. Generation and functional characterization of a novel genetically defined *Rev1*-KO and *Rev1*-Del mouse model.

A. genotyping PCR to determine whether mice feature the WT (lower band) or truncated allele (upper band).

B. Generation of *Rev1*-KO and *Rev1*-Del mice. CRISPR/Cas9 deleted part of exon 4 and 11. This prevents translation of a remaining product. In *Rev1*-Del mice, exon 4-13 are deleted, leaving exon 1-3, and exon 14-23.

C. *In silico* analysis with Benchling was performed to examine potential alternate splice products of *Rev1* in both mouse models, and whether and how these could be translated. Green cells refer to products that could potentially arise in *Rev1*-KO cells, green/blue cells refer to products that could appear in both mouse models.

D. Isolation of B cells from adult mice to examine somatic hypermutation and class switch recombination in B cells *ex vivo*. From adult mice, germinal center B cells were isolated from Peyer's patches from the intestine, followed by sequencing of the JH4 intronic region to determine the somatic hypermutation spectrum. Splenic B cells were purified by CD43 depletion, and these were cultured for 72 hours with LPS, with (IgG1) or without (IgG3) IL-4, after which class switching to IgG1 and IgG3 were determined.

E. Somatic hypermutation analysis of JH4 intron sequences from germinal center B cells shows a reduction in G/C>C/G transversions in *Rev1*-KO mice. The mutation frequencies of sequences from *Rev1*-KO mice were normalised to those of WT mice.

F. *In vitro* class switching recombination to IgG1 and IgG3 is reduced in *Rev1*-KO mice. A representative experiment of two independent experiments is shown. Significance was determined with the Mann-Whitney test; **$p<0.005$.

G. Somatic hypermutation base substitution frequencies of Peyer's patches B cells.

H Details of somatic hypermutation experiments.

Figure S2. Extended gating strategy for the hematopoietic system.

A.Table containing markers used for each hematopoietic subset.

B.Representative gating strategy including the CD34/CD48 markers used to distinguish MPP1-4 and HSCs.

C. H&E staining of the sternum of PBS and cisplatin treated mice.

Figure S3. Inactivation of p53 does not enhance replication stress or reduce growth speed of REV1-deficient cells*.*

A. Concentrations used for cisplatin and REV1i for synergy experiments in indicated cell lines.

B. Tumor growth *in vivo* of subcutaneously transplanted p53-KO lymphomas of the indicated genotypes.

C. Kaplan-Meier curve of mice bearing p53-KO lymphomas of the indicated genotypes. Survival was measured from the time that tumors reached 100 mm^3. Mice were sacrificed after the tumor reached 1500 mm^3.

D. Neutral comet assays in p53-WT primary MEFs (left panel) and p53 knockdown MEFs (right panel). Data from three independent experiments are pooled. One way Anova with multiple comparisons were used to determine significance. *$p<0.05$, ****$p<0.0001$.

Figure S4. TLS polymerase Kappa tolerates DNA interstrand crosslinks and is frequently deleted in a subset of human cancers. **Extended figure.**

A. Genetic map of *Polk* and *Ankdd1b* and *Col4a3bp*.

B. Whole genome sequencing track of WT parental lymphomas, showing reads of the *Polk* sequence and *Col4a3bp*.

C. Whole exome sequencing of the *Polk*-KO lymphoma depicting *Ankdd1b, Polk*, and *Col4a3bp*, indicating the loss of *Polk* in the *Polk*-KO line.

D. Z' values of all plates of the screen, indicating an excellent separation between positive and negative controls. Plates with a Z' lower than 0.45 were not taken along.

E. Example of two of the three replicate plates. Points indicate the effects of single drugs in each plate. Points along the diagonal line indicate that the drug has the same effect in the two indicated replicate plates.

F. Hits from the compound screen that are known to damage the DNA, and their clinical applications.

G. Steroid hormone hits from the compound screen.

H. Dose-effect response of two chosen steroid hormone related hits (Mifepristone, Wortmannin). Error bars denote standard deviation.

I. Structures of steroid hormone compounds detected as hits in the screen for one or more cell lines.

Fig S1

A

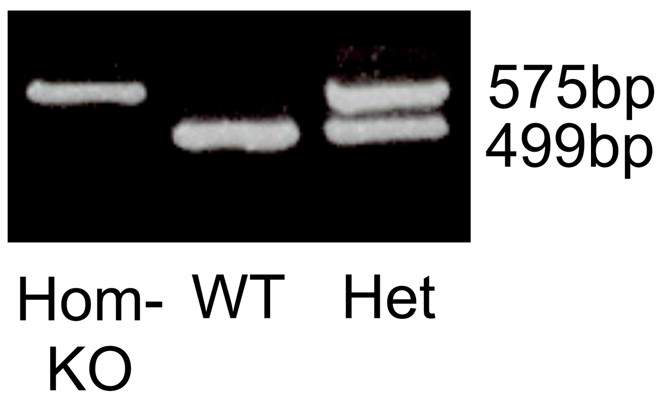

B

Rev1-KO out of frame deletion

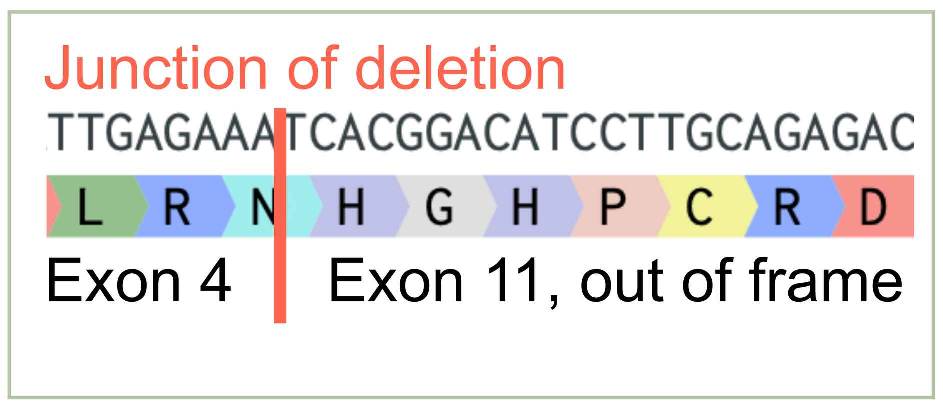

Rev1-Del out of frame transcript

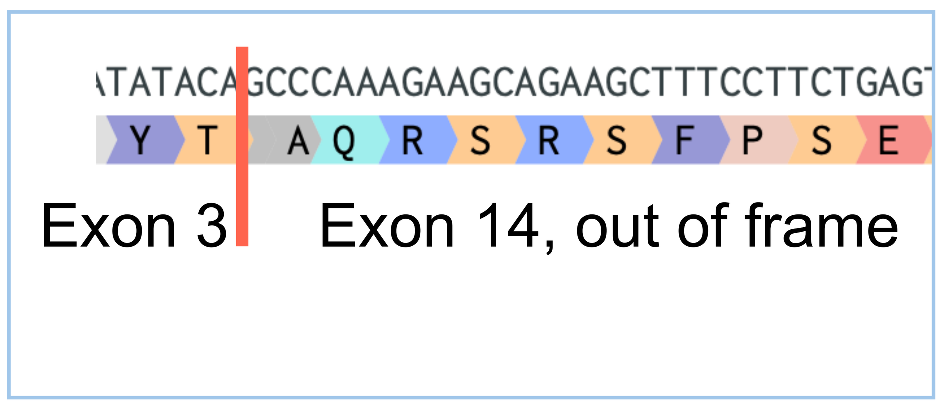

C

Hypothetical Rev1-Del transcript (exon-exon joining)	# of stop codons after junction	Molecular Weight (KDa)
**No alternative splicing 4-11**	**40**	**9.1**
2-12	35	5.4
3-12	1 (in frame)	77.0
3-13	1 (in frame)	72.5
**3-14**	**24**	**9.0**
3-15	27	6.9
3-16	23	7.9
3-17	1 (in frame)	48.2
3-18	18	6.9
3-19	9	7
3-20	1 (in frame)	28.4
3-21	6	8.3
3-22	1 (in frame)	15.2
3-23	3	6.9
2-14	1 (in frame)	59.8
2-15	25	3.2
2-16	1 (in frame)	49.8
2-17	20	8.8
2-18	1 (in frame)	36.7
2-19	1 (in frame)	28.4
2-20	13	3.4
2-21	1 (in frame)	16.1
2-22	7	3.6
2-23	2	3.7

Alternate products of *Rev1*-KO mice
Red denotes translation of hybrid exon 4-11

Alternate products of *Rev1*-KO, *Rev1*-Del mice
Red denotes translation of exon 3-14

D

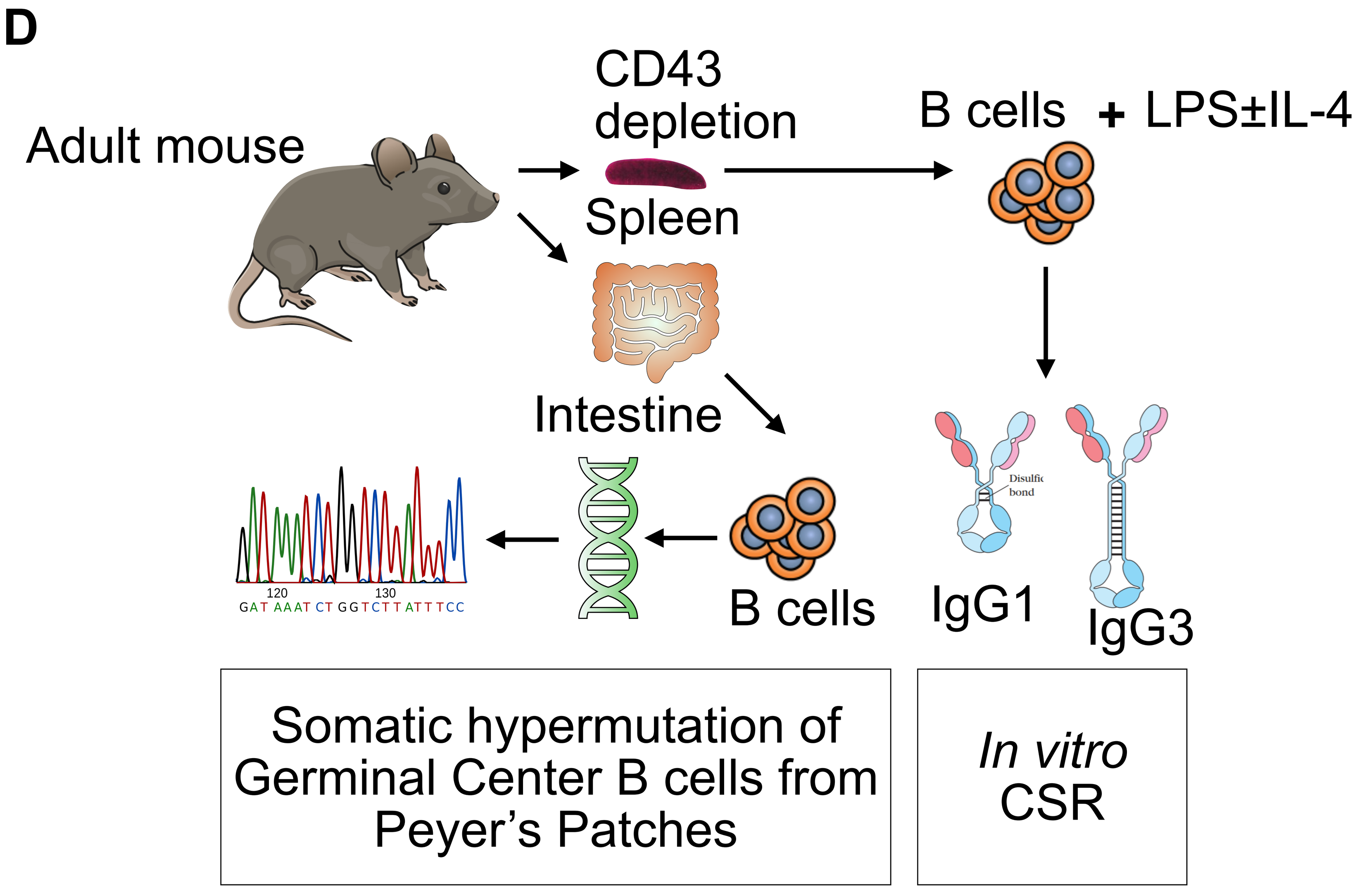

E

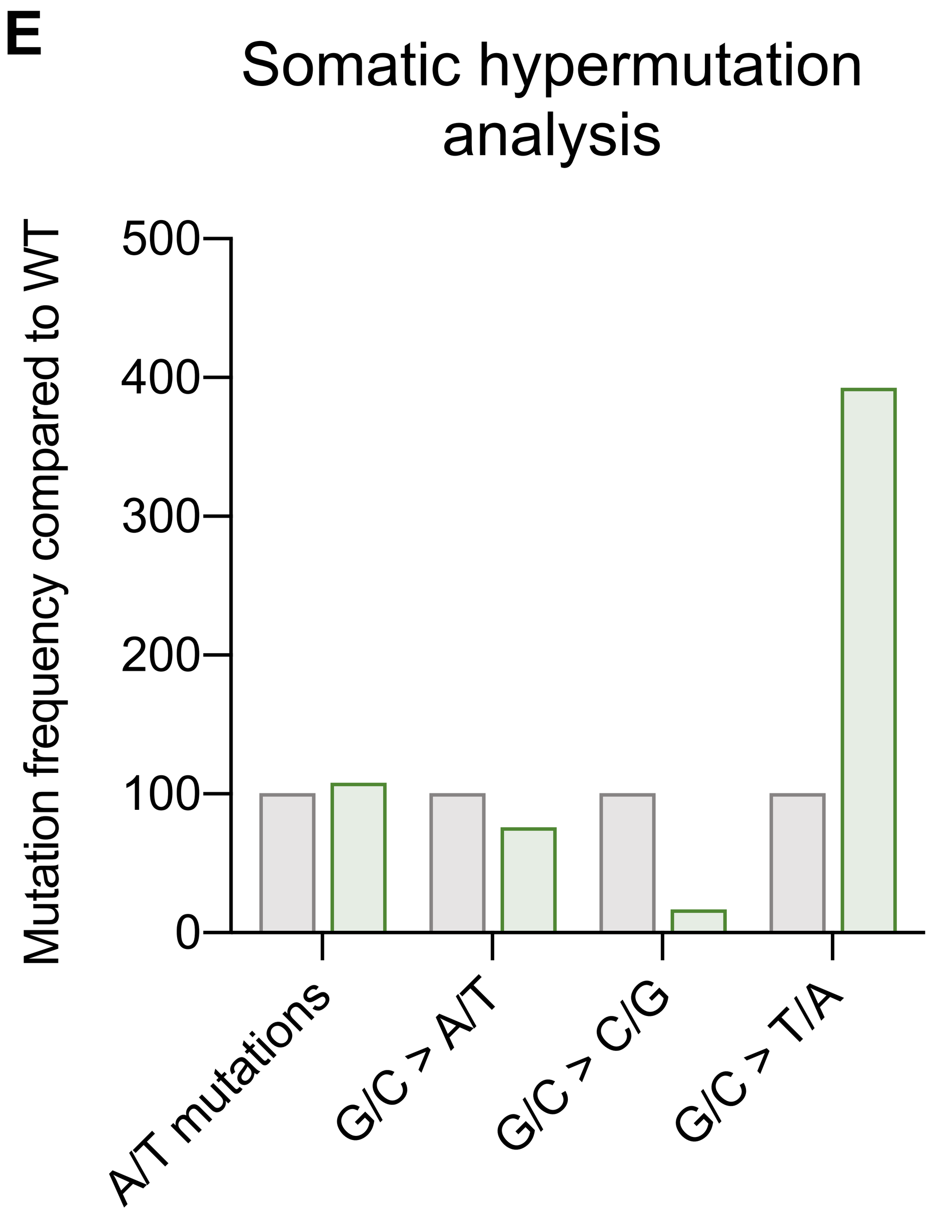

F

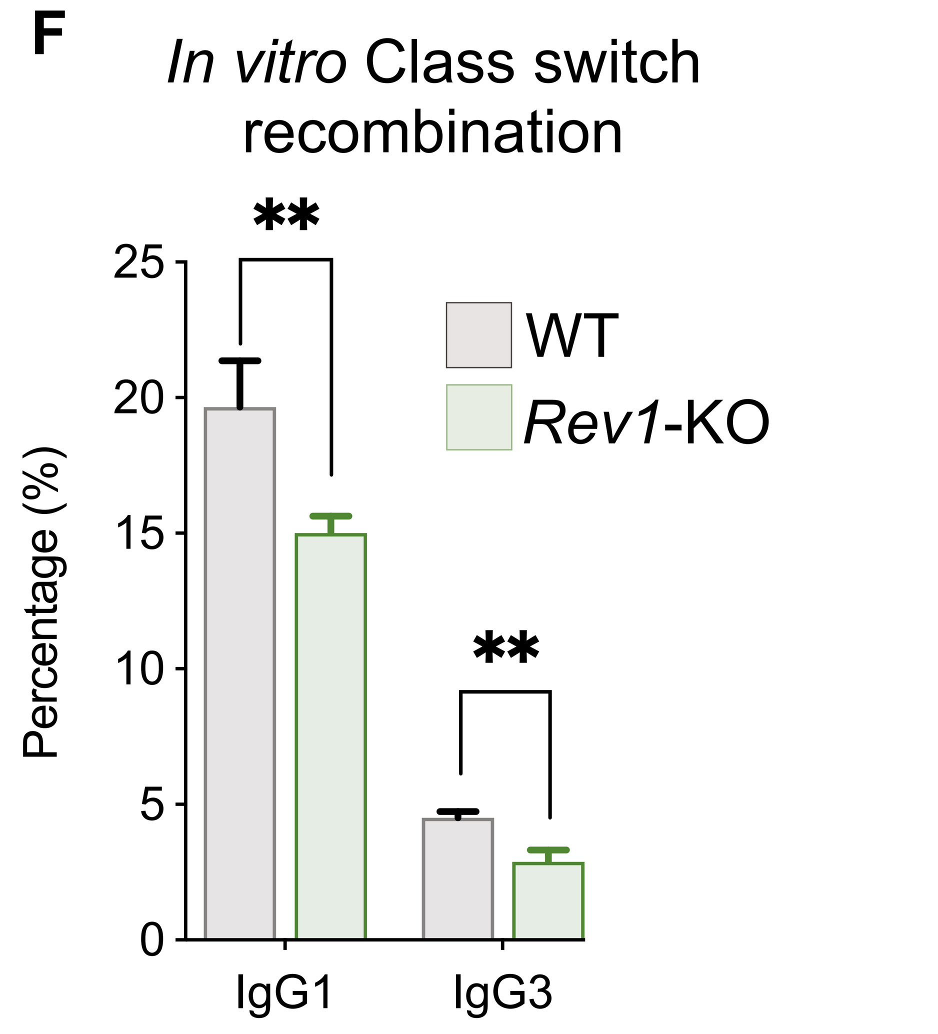

G

WT — To (columns), From (rows)

From \ To	A	G	C	T	%
A		16	7	10	33
G	10		6	6	22
C	3	4		9	16
T	12	7	10		29
					100

***Rev1*-KO** — To (columns), From (rows)

From \ To	A	G	C	T	%
A		23	10	15	48
G	10		1	5	16
C	4	0		8	12
T	8	4	12		24
					100

H

	WT	*Rev1*-KO
# mice	5	3
# mutated sequences	67	62
Total mutations	783	389
Total bp sequenced	34259	31363
mutations/bp (%)	2.3	1.2

Fig S2

A

Subset	Subpopulation	Markers
LK	LSK, Myeloid progenitors	Lin$^-$, cKit$^+$
LSK	HSC, MPP1-4	Lin$^-$, cKit$^+$, Sca-1$^+$
LKS$^-$ (Myeloid progenitors)	GMP, CMP, MEP	Lin$^-$, cKit$^+$, Sca-1$^-$
CLP progenitors	CLP	Lin$^-$, cKit^{intermediate}, Sca-1^{intermediate}
MPP1 (multipotent progenitor 1)		Lin$^-$, cKit$^+$, Sca-1$^+$, CD135$^-$, CD150$^+$, CD34$^+$, CD48$^-$
MPP2 (multipotent progenitor 2)		Lin$^-$, cKit$^+$, Sca-1$^+$, CD135$^-$, CD150$^+$, CD34$^+$, CD48$^+$
MPP3 (multipotent progenitor 3)		Lin$^-$, cKit$^+$, Sca-1$^+$, CD135$^-$, CD150$^-$, CD34$^+$, CD48$^+$
MPP4 (multipotent progenitor 4)		Lin$^-$, cKit$^+$, Sca-1$^+$, CD135$^+$, CD150$^-$, CD34$^+$, CD48$^+$
HSC (hematopoietic stem cells)		Lin$^-$, cKit$^+$, Sca-1$^+$, CD135$^-$, CD150$^+$, CD34$^-$, CD48$^-$
CLP (common lymphoid progenitor)		Lin$^-$, cKit^{intermediate}, Sca-1^{intermediate}, CD127$^+$, CD135$^+$
GMP (granulocyte/macrophage progenitor)		Lin$^-$, cKit$^+$, Sca-1$^-$,CD16/32$^+$, CD34$^+$
CMP (common myeloid progenitor)		Lin$^-$, cKit$^+$, Sca-1$^-$,CD16/32^{intermediate}, CD34^{intermediate}
MEP (megakaryocyte erythrocyte progenitor)		Lin$^-$, cKit$^+$, Sca-1$^-$,CD16/32$^-$, CD34$^-$

B

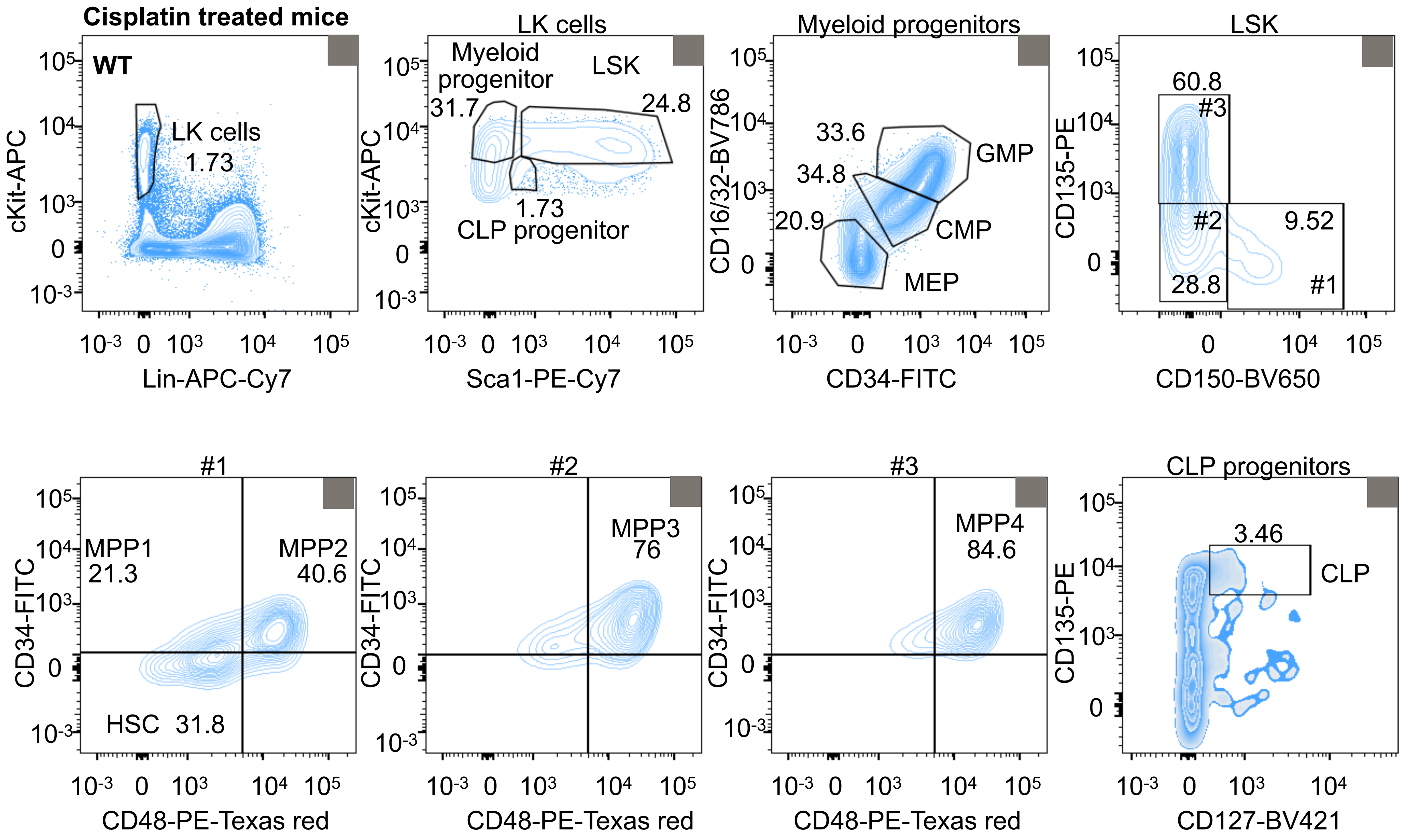

C

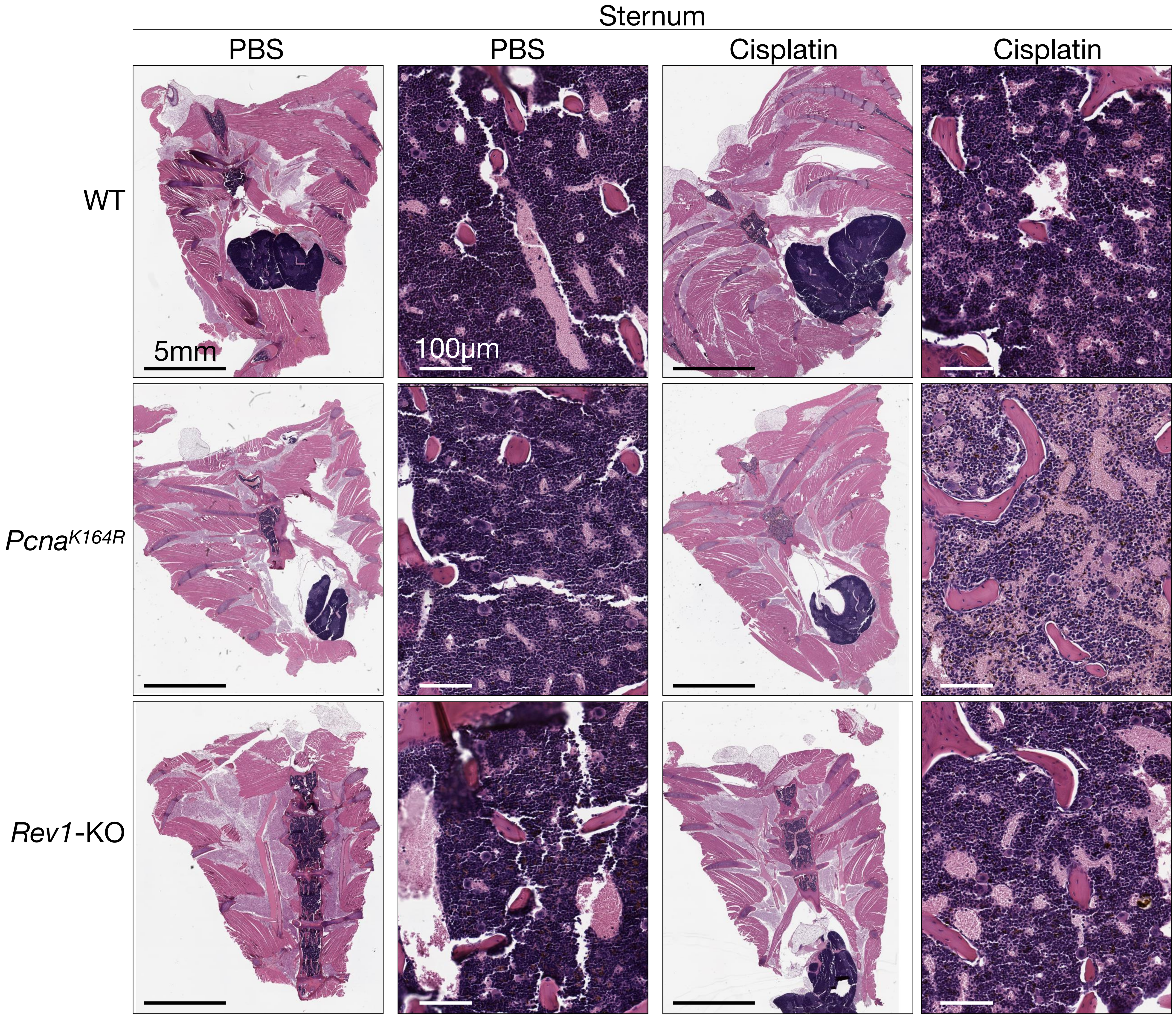

Fig S3

A

Cell line	Concentration cisplatin (μM)	Titrated against REV1i concentrations (μM)
LNCaP	1.17, 2.54, 4.69, 9.38, 18.8	0.3125, 0.6125, 1.25, 2.5, 5
22rv1	0.3125, 0.6125, 1.25, 2.5, 5	0.3125, 0.6125, 1.25, 2.5, 5
P53-deficient WT lymphoma	0.05, 0.1, 0.2, 0.4, 0.8	0.078, 0.156, 0.313, 0.613, 1.25
P53-deficient *Pcna*^{K164R} lymphoma	0.05, 0.1, 0.2, 0.4, 0.8	0.078, 0.156, 0.313, 0.613, 1.25
P53-deficient *Rev1*-KO lymphoma	0.05, 0.1, 0.2, 0.4, 0.8	0.078, 0.156, 0.313, 0.613, 1.25
MCF7 p53-WT	0.3125, 0.6125, 1.25, 2.5, 5	0.078, 0.156, 0.313, 0.613, 1.25
MCF7 p53-KO	0.125, 0.25, 0.5, 1, 2	0.078, 0.156, 0.313, 0.613, 1.25
HCT116 p53-WT	0.9375, 1.875, 3.75, 7.5, 15	0.21875, 0.4375, 0.875, 1.75, 3.5
HCT116 p53-KO	0.9375, 1.875, 3.75, 7.5, 15	0.21875, 0.4375, 0.875, 1.75, 3.5
A375	0.28, 0.56, 1.11, 2.25, 4.5	0.28, 0.56, 1.11, 2.25, 4.5
Skov3	0.28, 0.56, 1.11, 2.25, 4.5	0.28, 0.56, 1.11, 2.25, 4.5
WT MEF	0.28, 0.56, 1.11, 2.25, 4.5	0.28, 0.56, 1.11, 2.25, 4.5
Pcna^{K164R} MEF	0.28, 0.56, 1.11, 2.25, 4.5	0.28, 0.56, 1.11, 2.25, 4.5
Rev1-KO MEF	0.28, 0.56, 1.11, 2.25, 4.5	0.28, 0.56, 1.11, 2.25, 4.5

B

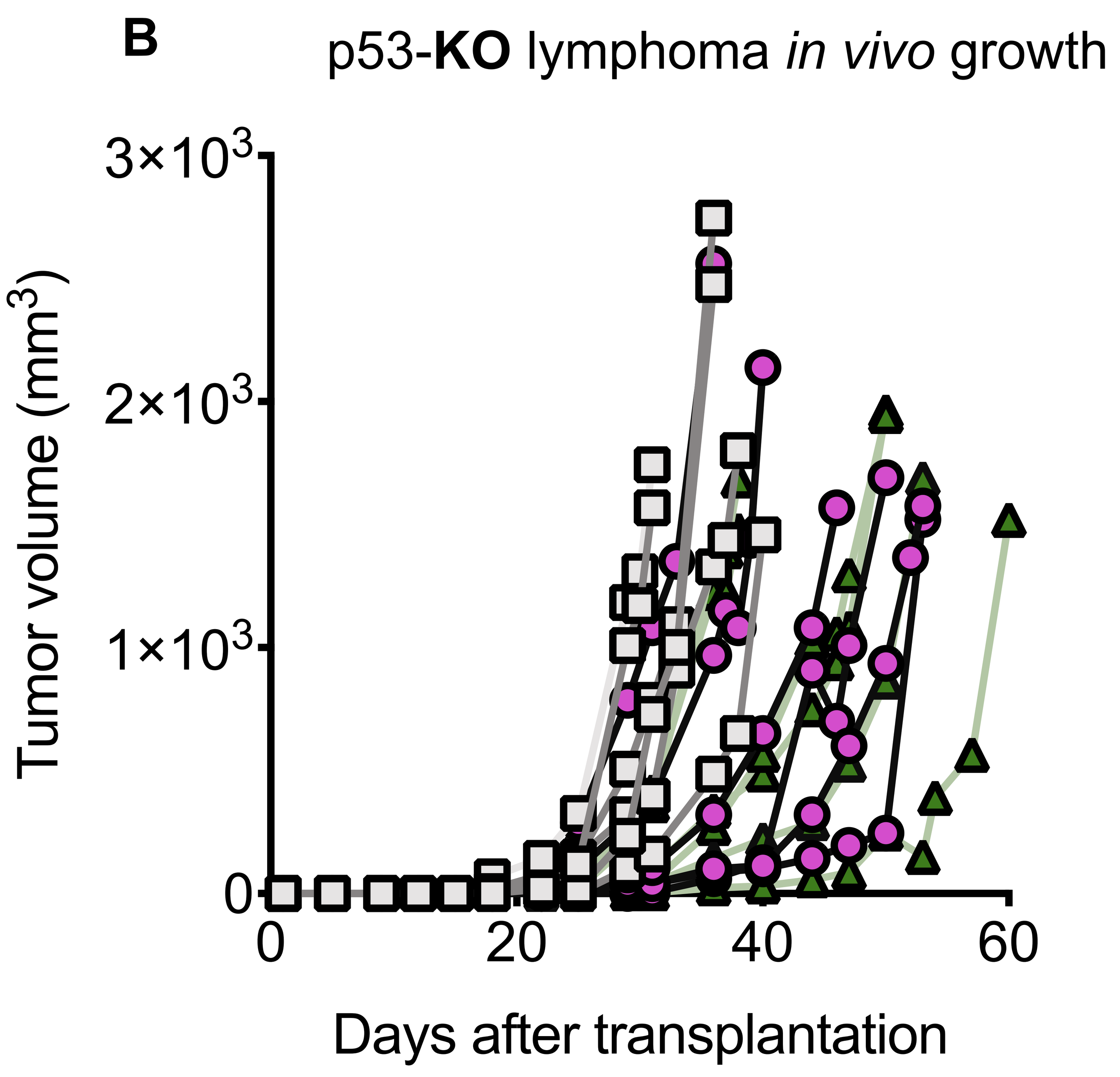

C

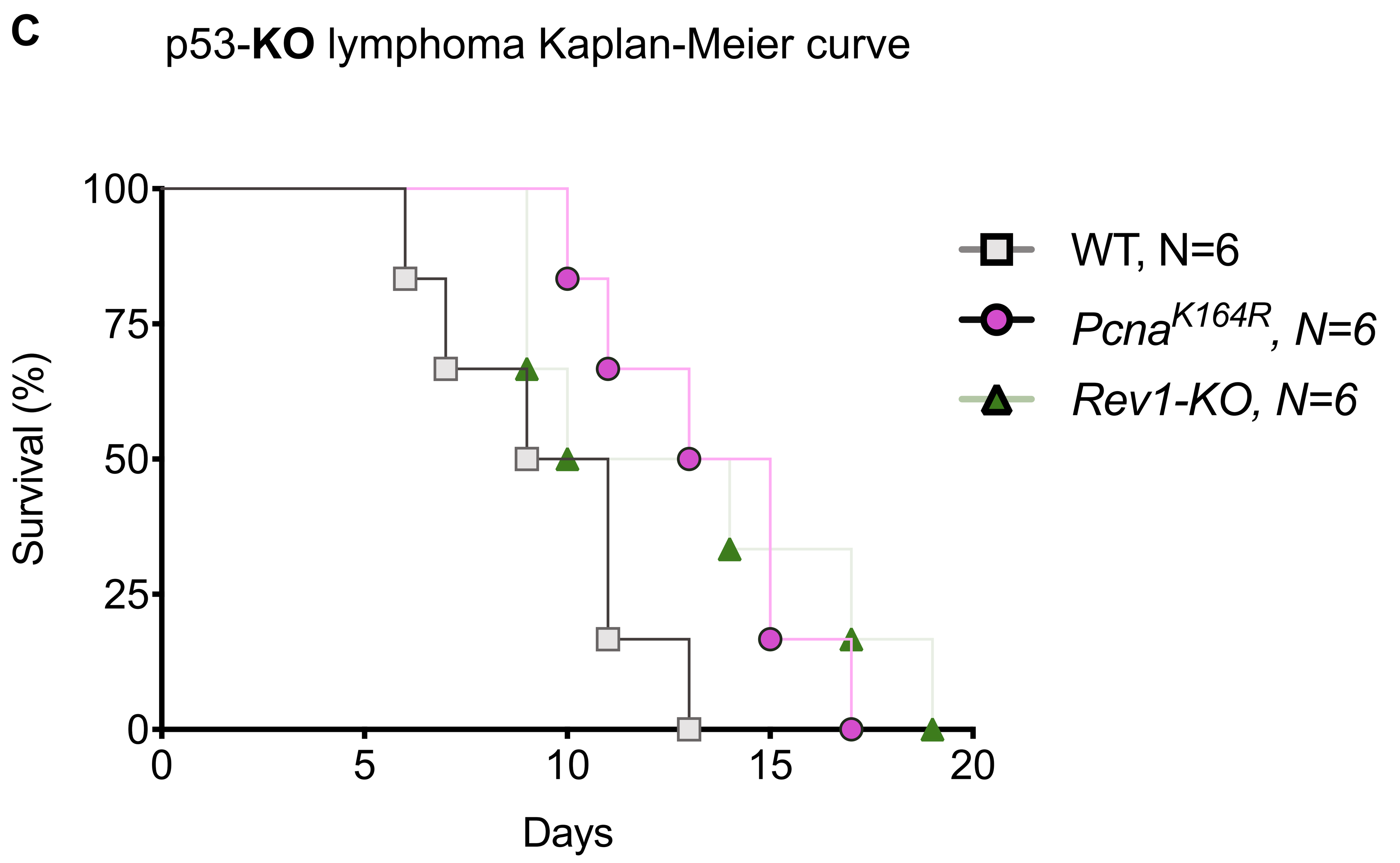

D

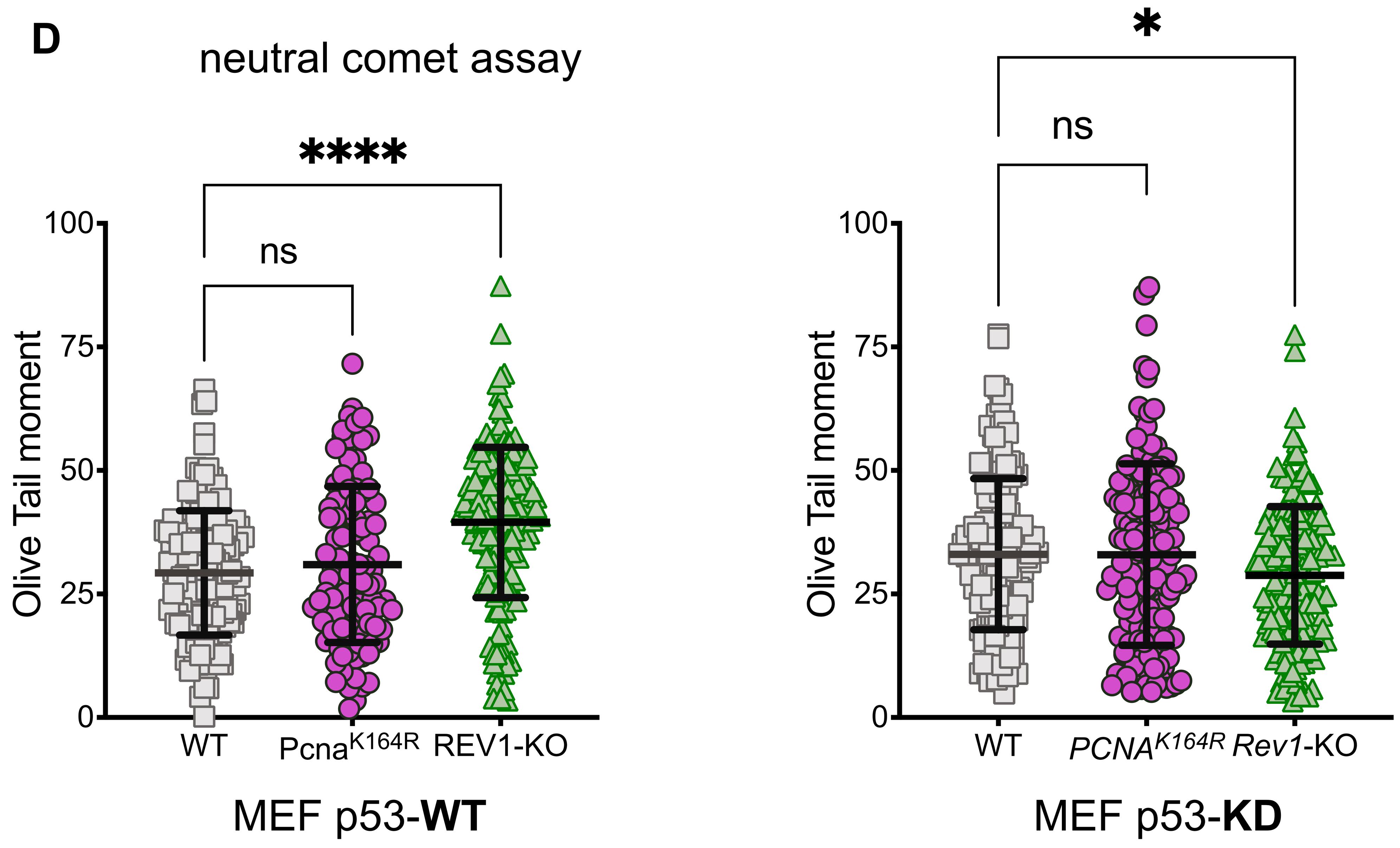

Fig S4

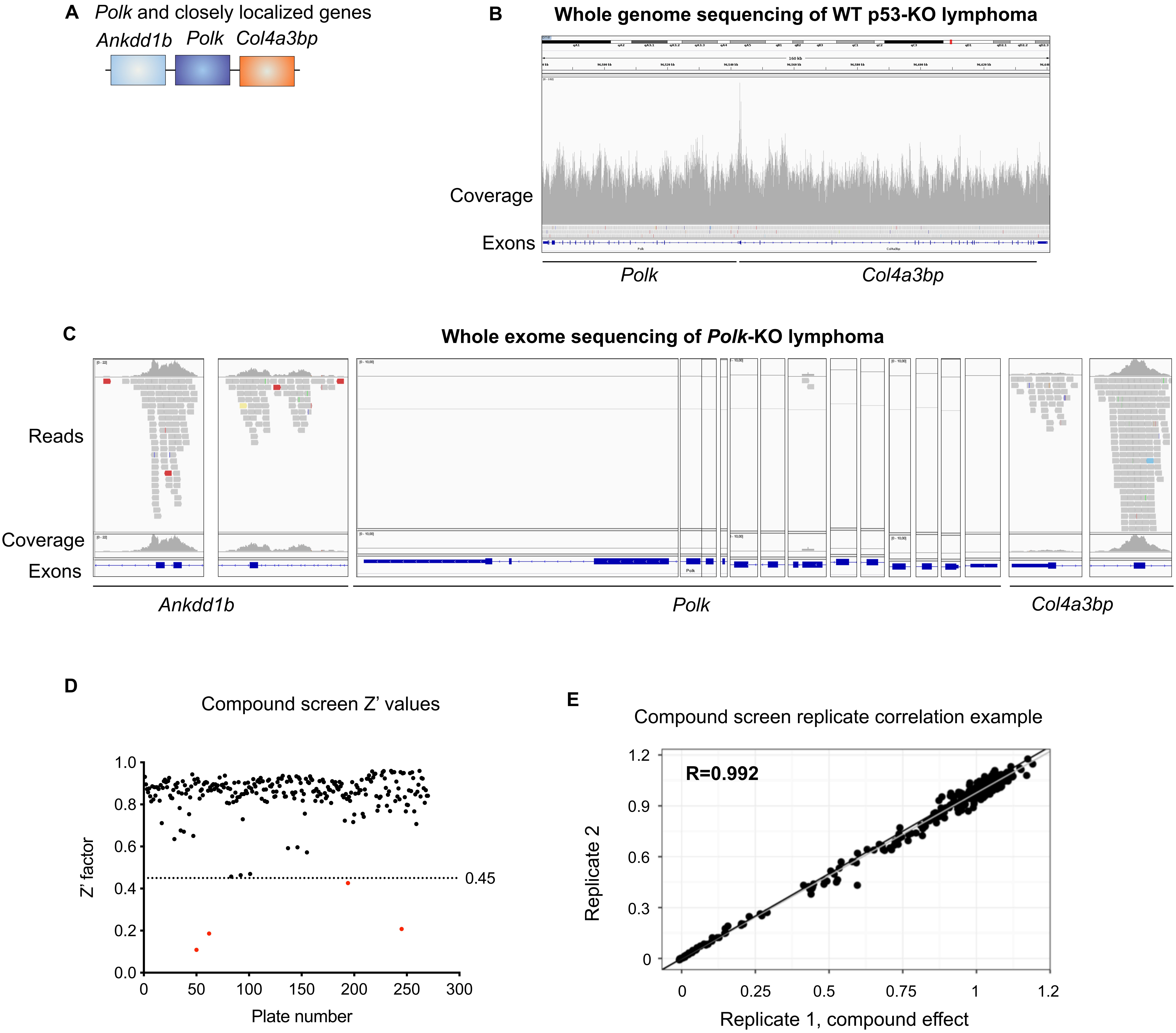

F

DNA damaging agents/modifiers	Effect on DNA	Clinical use
Nitrogen Mustard	Alkylation and ICL	Lung cancer, breast cancer, T-cell lymphoma, Hodgkin disease, non-Hodgkin lymphoma
Melphalan	Alkylation and ICL	Myeloma, ovarian cancer, melanoma
5-Bromo-2'-deoxyuridine	Incorporation during S-phase	NA
5-Fluorouracil	Inhibits thymidilate synthase	Gastrointestinal carcinoma
RITA (NSC 652287)	Formation of DNA-protein crosslinks and DNA ICLs	NA
Chlorambucil	Alkylation and ICL	Chronic lymphocytic leukemia
Nimustine hydrochloride	Alkylation and ICL	Brain cancer

Continues on next page

Fig S4

G

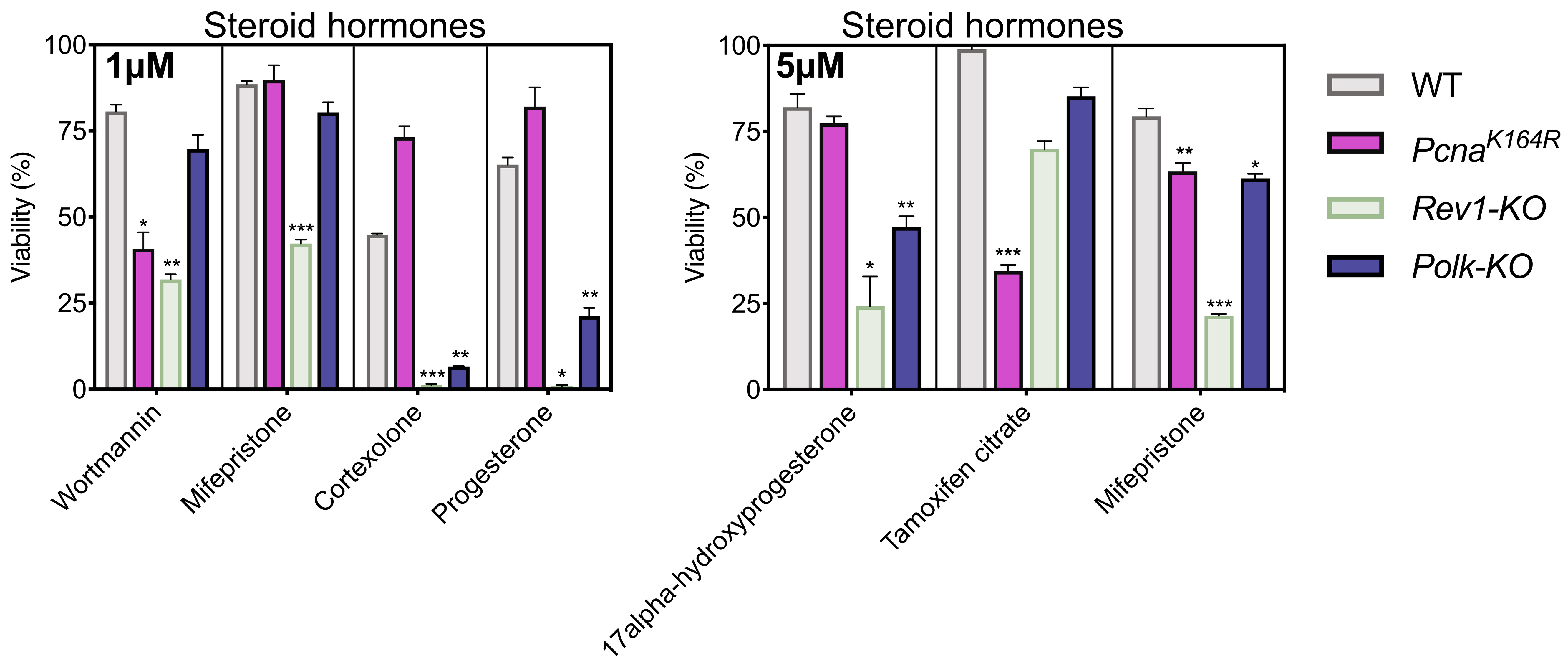

H

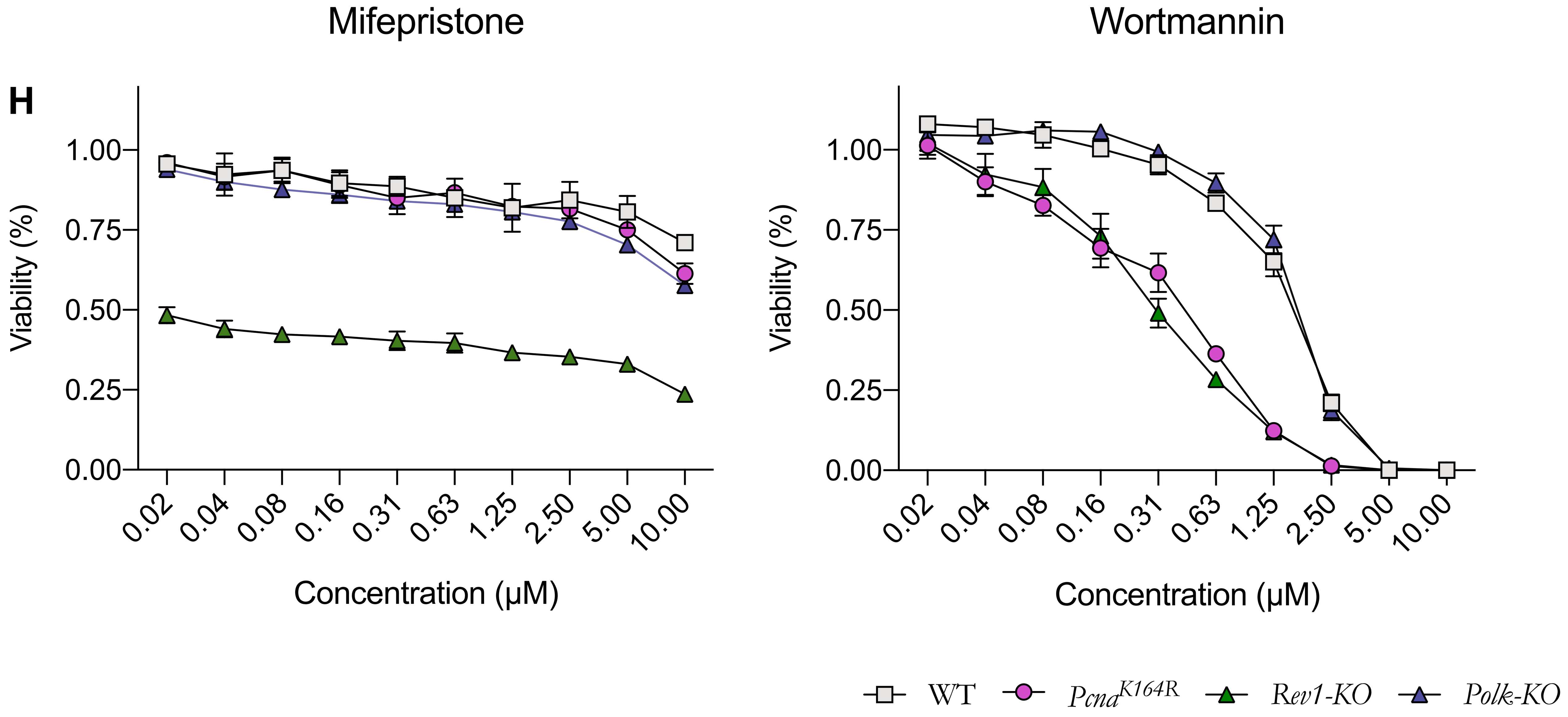

I **Steroid hormones structures**

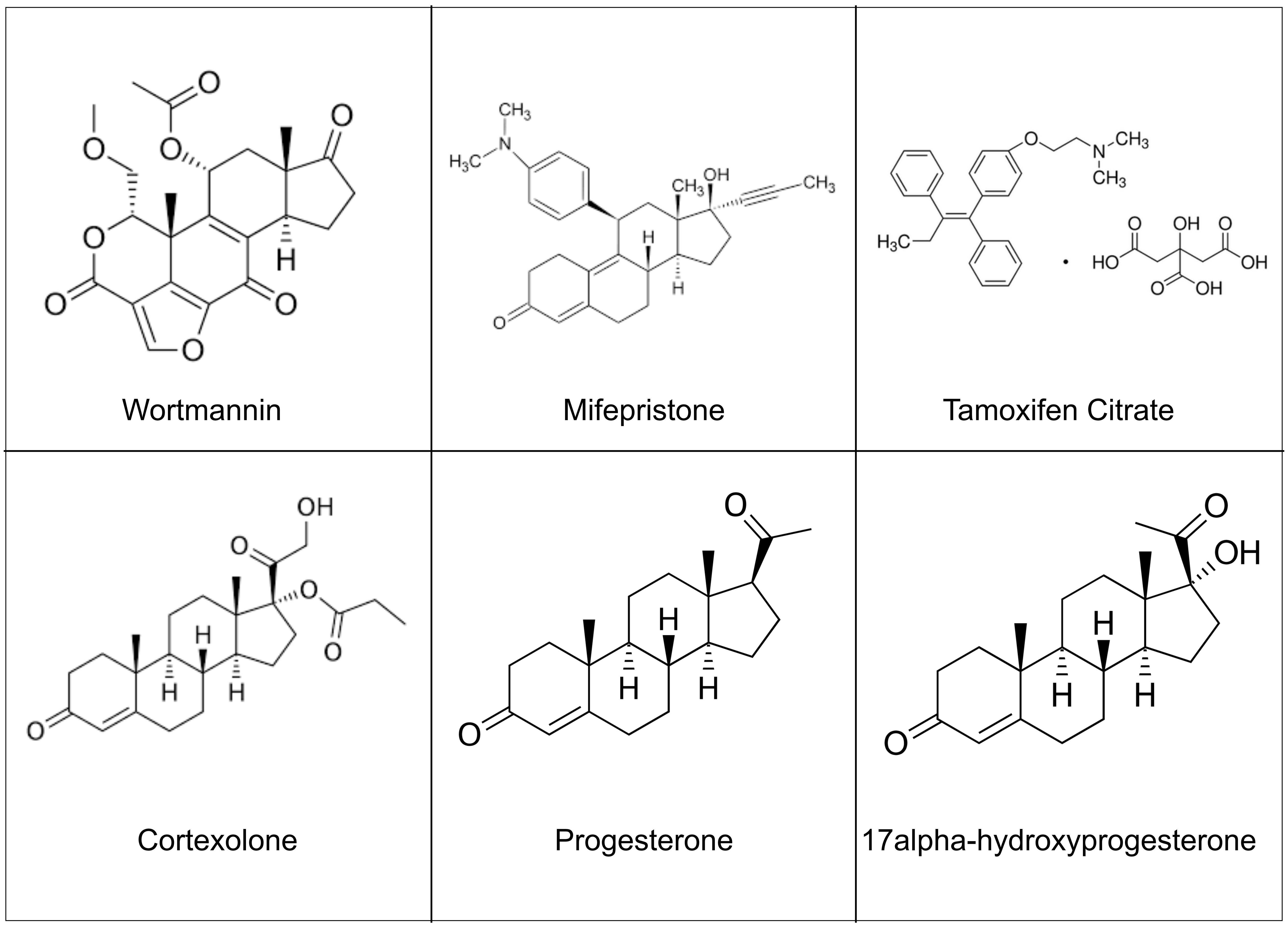

Continues from previous page

Table S5: Oligonucleotide sequences used in the study, ordered either from Integrated DNA technologies (IDT) or Termo Fisher.

**Oligonucleotides for PCRs and gRNAs**	**Sequence (5'-3')**	**Purpose**
PCNA genotype FWD	TGCAAGTGGAGAGCTTGGCAATG	Determine whether mice carry *Pcna*^{K164} WT allele, or mutant *Pcna*^{K164R} allele.
PCNA genotype REV	CTTTCCAAATGCTACCTGTggcg	Determine whether mice carry *Pcna*^{K164} WT allele, or mutant *Pcna*^{K164R} allele.
REV1-KO genotype FWD	GGCAACATGGCCAAGAAGAAC	Determine whether mice carry *Rev1* WT or KO allele. Identical for mice, MEFs, and lymphoma
REV1-KO genotype REV	TTATTCAGCTTGGCGAGCGCTTTTG	Determine whether mice carry *Rev1* WT or KO allele. Identical for mice, MEFs, and lymphoma
REV1-KO genotype INT	ACTCAGTCAGCAGACACATGC	Determine whether mice carry *Rev1* WT or KO allele. Identical for mice, MEFs, and lymphoma
REV1-Del genotype 5'Flox FWD	GATGGCTCACAGGGTAAAGATGC	Determine whether the LoxP site is still detected in *Rev1*-Del mice.
REV1-Del genotype 5'Flox REV	TTTATGCTGTCACTGACCTGTTGC	Determine whether the LoxP site is still detected in *Rev1*-Del mice.
REV1-Del genotype Del FWD	GATGGCTCACAGGGTAAAGATGC	Determine whether the *Rev1* deletion has occurred *Rev1*-Del mice.
REV1-Del genotype Del REV	CCACATTTCTGCCTGAAACCC	Determine whether the *Rev1* deletion has occurred *Rev1*-Del mice.
POLK-KO genotype FWD	ACTCACTCACACCTCCGCTA	Determine whether mice carry *Polk* WT or KO allele.
POLK-KO genotype REV	CACAACCAATAACCAAAGGACCA	Determine whether mice carry *Polk* WT or KO allele.
POLK-KO genotype INT	ACCCTACCCCCAAACACTCAAAAT	Determine whether mice carry *Polk* WT or KO allele.
POLK-KO genotype INT-FWD exon 12	ATAGGCTTCTTTCTCCCTCCCT	Determine whether mice carry *Polk* WT or KO allele. Second primer pair.
POLK-KO genotype INT-REV exon 12	AGGCTAGAAGCTTCTGGGACTA	Determine whether mice carry *Polk* WT or KO allele. Second primer pair.
Mouse PCNA gRNA1 Intron 1	TAGTAAGGGGGCGTCCAGTT	Used to remove exon 2-4 of *Pcna* in p53-KO lymphoma
Mouse PCNA gRNA2 Intron 5	GAATTTTGGACATGCTAGGG	Used to remove exon 2-4 of *Pcna* in p53-KO lymphoma
POLK gRNA1 upstream first exon	GTTCTCACGTCCCGGCTCGC	Used to remove entire *Polk* gene from p53-KO lymphoma
POLK gRNA2 downstream last exon	GCCATACAAGGTCGGTTCTA	Used to remove entire *Polk* gene from p53-KO lymphoma
REV1 gRNA1 exon 4	AGAAATCTAATGATGTTGCATGG	Used to remove *Rev1* exon 4-11 from mice and p53-KO lymphomas.
REV1 gRNA2 exon 11	TGAAGCACTGATTGACGTCACGG	Used to remove *Rev1* exon 4-11 from mice and p53-KO lymphomas.
REV1 gRNA1 intron 3	CCCTAGCCCTTTAATATAACAGG	Used to introduce LoxP site into intron 3 in *Rev1*-Del mice.
REV1 gRNA2 intron 13	CAAACGTGCATTCGAGGGACAGG	Used to introduce LoxP site into intron 13 in *Rev1*-Del mice.
REV1 cDNA exon 2-3 FWD	GATGGCTGGGAAAAATGGGG	Used to determine levels of *Rev1* in MEFs.
REV1 cDNA exon 2-3 REV	GATCTCGCTCCTGGAAGATG	Used to determine levels of *Rev1* in MEFs.
REV1 cDNA exon 17-18 FWD	CCCAGAGCCTCAAGAACCT	Used to determine levels of *Rev1* in MEFs.
REV1 cDNA exon 17-18 REV	TGTACTGGTTGGCTGCTGA	Used to determine levels of *Rev1* in MEFs.
GAPDH cDNA FWD	CAATGACCCCTTCATTGACC	Used as normalization controls.
GAPDH cDNA REV	GATCTCGCTCCTGGAAGATG	Used as normalization controls.
smartPOOL hREV1	SMARTpool: ON-TARGETplus REV1 siRNA; cat L-008234-00-0020	Used to knock down *Rev1* in human cancer cells.
smartPOOL siNon-Targeting	ON-TARGETplus Non-targeting Pool; cat D-001810-10-20	Used as non-targeting controls.

Table S6: Mouse models used in this study.

**Model organisms: mice**	**Source**
Nude mice (NMRI Rj)	Janvier
C57Bl/6J	Janvier
Pcna$^{K164R\ mice}$	Langerak *et al.*(14)
Rev1-KO mice	Described in this study
Rev1-Del mice	Described in this study

Table S7: Bacteria used in this study.

**Model organisms: bacterial strains**	**Source**
Competent DH5a	In-house

Table S8: Cell models used in this study

**Cell lines**	**Source**
P53-KO lymphoma	Buoninfante *et al.*(18)
LNCaP	Zwart lab, in-house
22Rv1	Zwart lab, in-house
PreB cells	In-house
Mouse Embryonic Fibroblasts	In-house
HCT116, P53-wt/ko	Wang *et al.(2*0)
MCF-7, P53-wt/ko	Wellenstein *et al,* (19)
A375	Bernard's Lab, in-house
SKOV3	Bernard's Lab, in-house
HEK 293T cells	Jonker's Lab, in-house
J558-IL-7 producer cells	Rolink (15)

Table S9: Vectors used in this study.

Recombinant DNA	Source
pCL-ECO	In-house, see (39)
pMX-IRES-GFP	In-house, see (39)
pMX-mPOLK-GFP	In-house, see (39)
pX330-puro	In house, from Henri
pX333-mCherry	In-house, see (18)
pMX-eGFP	Amaxa, Lonza

Table S10: Reagents used in this study.

Reagents	Source
JH-RE-06 (REV1 inhibitor)	MedChem express; cat HY-126214
Cisplatin	In-house
RNAImax	Thermofisher; cat 13778030
Nucleofector Cuvettes	Sopachem BV, mirus; cat. MIR 50121

Table S11: Antibodies used in this study.

Antibodies used in study	Manufacturer	Dilution
cKit-APC, 2B8	eBioscience	1:200
Streptavidin-APC-Cy7	Southern Biotech	1:200
CD135-APE, A2F10	Biolegend	1:200
CD48-PE-Dazzle, HM48-1	Biolegend	1:200
7AAD-PE-Cy5	Biolegend	1:200
Sca-1-PE-Cy7, D7	Biolegend	1:200
CD34-FITC, RAM34	Biolegend	1:100
CD127-BV421, A7R34	Biolegend	1:200
CD150-BV650, TC15-12F12.2	Biolegend	1:200
CD16/32-BV785, 2.4G2	BD Bioscience	1:200
Lineage cell detection cocktail biotin	Miltenyi	1:40
UltraComp eBeads	Invitrogen	1 drop/5 samples
Rev1, OTIE12	Invitrogen	1:200-1:500
GFP, 3E6	Invitrogen	1:1000
ß-Actin, AC-15	Sigma Aldrich	1:5000
PCNA (PC-10)	Santa Cruz	1:1000
Actin, clone C4	Merck Milipore	1:10000
FLAG monoclonal, F3165	Sigma-Aldrich	1:1000
HorseRadish	Dako	1:2500
Horse Radish Peroxidase	Dako	1:2500
680-RD, anti-mouse	LiCor	1:10000
800-CW, anti-mouse	LiCor	1:10000
PNA-FITC	Vector Laboratories	1:400
CD19-APC, 1D3	BD pharmingen	1:300
CD95-PE, Jo2	BD pharmingen	1:200
CD43-Biotin, S7	BD biosciences	1:100

Table S12: PCR settings

PCR settings	Time
95ºC	3min
75ºC	5min
72ºC	1.5min
Melting - 95ºC (30-40 cycles)	30sec
Annealing - 63ºC (30-40 cycles)	30sec
Extension - 72ºC (30-40 cycles)	45sec
Final extension - 72ºC	10min
Storage – 4ºC-12ºC	Until usage
