# Supplementary material for: Division of labor within the DNA damage tolerance system reveals non-epistatic and clinically actionable targets for precision cancer medicine
Source: Nucleic Acids Res. 2022 Jul 12;50(13):7420–35. doi: 10.1093/nar/gkac545 (PMC9303390; doi:10.1093/nar/gkac545)
Supplement: gkac545_Supplemental_Files [file gkac545_supplemental_files.zip › Suppl.tables_NAR_HJ_120422.docx]

**Supplementary tables, belonging with materials and methods:**

**Table S1:** Oligonucleotide sequences used in the study, ordered either from Integrated DNA technologies (IDT) or Termo Fisher.

| **Oligonucleotides for PCRs and gRNAs** | **Sequence (5’-3’)** | **Purpose** |
| --- | --- | --- |
| PCNA genotype FWD | TGCAAGTGGAGAGCTTGGCAATG | Determine whether mice carry *Pcna^K164^* WT allele, or mutant *Pcna^K164R^* allele. |
| PCNA genotype REV | CTTTCCAAATGCTACCTGTggcg | Determine whether mice carry *Pcna^K164^* WT allele, or mutant *Pcna^K164R^* allele. |
| REV1-KO genotype FWD | GGCAACATGGCCAAGAAGAAC | Determine whether mice carry *Rev1* WT or KO allele. Identical for mice, MEFs, and lymphoma |
| REV1-KO genotype REV | TTATTCAGCTTGGCGAGCGCTTTTG | Determine whether mice carry *Rev1* WT or KO allele. Identical for mice, MEFs, and lymphoma |
| REV1-KO genotype INT | ACTCAGTCAGCAGACACATGC | Determine whether mice carry *Rev1* WT or KO allele. Identical for mice, MEFs, and lymphoma |
| REV1-Del genotype 5’Flox FWD | GATGGCTCACAGGGTAAAGATGC | Determine whether the LoxP site is still detected in *Rev1*-Del mice. |
| REV1-Del genotype 5’Flox REV | TTTATGCTGTCACTGACCTGTTGC | Determine whether the LoxP site is still detected in *Rev1*-Del mice. |
| REV1-Del genotype Del FWD | GATGGCTCACAGGGTAAAGATGC | Determine whether the *Rev1* deletion has occurred *Rev1*-Del mice. |
| REV1-Del genotype Del REV | CCACATTTCTGCCTGAAACCC | Determine whether the *Rev1* deletion has occurred *Rev1*-Del mice. |
| POLK-KO genotype FWD | ACTCACTCACACCTCCGCTA | Determine whether mice carry *Polk* WT or KO allele. |
| POLK-KO genotype REV | CACAACCAATAACCAAAGGACCA | Determine whether mice carry *Polk* WT or KO allele. |
| POLK-KO genotype INT | ACCCTACCCCCAAACACTCAAAAT | Determine whether mice carry *Polk* WT or KO allele. |
| POLK-KO genotype INT-FWD exon 12 | ATAGGCTTCTTTCTCCCTCCCT | Determine whether mice carry *Polk* WT or KO allele. Second primer pair. |
| POLK-KO genotype INT-REV exon 12 | AGGCTAGAAGCTTCTGGGACTA | Determine whether mice carry *Polk* WT or KO allele. Second primer pair. |
| Mouse PCNA gRNA1 Intron 1 | TAGTAAGGGGGCGTCCAGTT | Used to remove exon 2-4 of *Pcna* in p53-KO lymphoma |
| Mouse PCNA gRNA2 Intron 5 | GAATTTTGGACATGCTAGGG | Used to remove exon 2-4 of *Pcna* in p53-KO lymphoma |
| POLK gRNA1 upstream first exon | GTTCTCACGTCCCGGCTCGC | Used to remove entire *Polk* gene from p53-KO lymphoma |
| POLK gRNA2 downstream last exon | GCCATACAAGGTCGGTTCTA | Used to remove entire *Polk* gene from p53-KO lymphoma |
| REV1 gRNA1 exon 4 | AGAAATCTAATGATGTTGCATGG | Used to remove *Rev1* exon 4-11 from mice and p53-KO lymphomas. |
| REV1 gRNA2 exon 11 | TGAAGCACTGATTGACGTCACGG | Used to remove *Rev1* exon 4-11 from mice and p53-KO lymphomas. |
| REV1 gRNA1 intron 3 | CCCTAGCCCTTTAATATAACAGG | Used to introduce LoxP site into intron 3 in *Rev1*-Del mice. |
| REV1 gRNA2 intron 13 | CAAACGTGCATTCGAGGGACAGG | Used to introduce LoxP site into intron 13 in *Rev1*-Del mice. |
| FLAG Tag N-Terminal primer FWD | tttttGGATCCGCCGCCACCATGGACTACAAAGACGATGACGACAAGAGGCGAGGTGGATGGCGG | Used to add FLAG tag to *Rev1* transcript from *Rev1*-KO MEFs. |
| FLAG Tag N-Terminal primer Rev | aaaaaGCGGCCGCTCAGGTCACTTTCAGTGTGC | Used to add FLAG tag to *Rev1* transcript from *Rev1*-KO MEFs. |
| FLAG Tag C-Terminal primer FWD | tttttGGATCCGCCGCCACCATGAGGCGAGGTG | Used to add FLAG tag to *Rev1* transcript from *Rev1*-KO MEFs. |
| FLAG Tag C-Terminal primer Rev | AAAAAGCGGCCGCTCACTTGTCGTCATCGTCTTTGTAGTCGGTCACTTTCAGTGTGCTTCCATA | Used to add FLAG tag to *Rev1* transcript from *Rev1*-KO MEFs. |
| REV1 cDNA exon 2-3 FWD | GATGGCTGGGAAAAATGGGG | Used to determine levels of *Rev1* in MEFs. |
| REV1 cDNA exon 2-3 REV | GATCTCGCTCCTGGAAGATG | Used to determine levels of *Rev1* in MEFs. |
| REV1 cDNA exon 17-18 FWD | CCCAGAGCCTCAAGAACCT | Used to determine levels of *Rev1* in MEFs. |
| REV1 cDNA exon 17-18 REV | TGTACTGGTTGGCTGCTGA | Used to determine levels of *Rev1* in MEFs. |
| GAPDH cDNA FWD | CAATGACCCCTTCATTGACC | Used as normalization controls. |
| GAPDH cDNA REV | GATCTCGCTCCTGGAAGATG | Used as normalization controls. |
| smartPOOL hREV1 | SMARTpool: ON-TARGETplus REV1 siRNA; cat L-008234-00-0020 | Used to knock down *Rev1* in human cancer cells. |
| smartPOOL siNon-Targeting | ON-TARGETplus Non-targeting Pool; cat D-001810-10-20 | Used as non-targeting controls. |

**Table S2:** Mouse models used in this study.

| **Model organisms: mice** | **Source** |
| --- | --- |
| Nude mice (NMRI Rj) | **Janvier** |
| C57Bl/6J | **Janvier** |
| *Pcna^K164R^* mice | Langerak *et al***.**(1) |
| *Rev1*-KO mice | Described in this study |
| *Rev1-*Del mice | Described in this study |

**Table S3:** Bacteria used in this study.

| **Model organisms: bacterial strains** | **Source** |
| --- | --- |
| Competent DH5a | **In-house** |

**Table S4:** Cell models used in this study

| **Cell lines** | **Source** |
| --- | --- |
| P53-KO lymphoma | Buoninfante *et al.*^18^ |
| LNCaP | Zwart lab, in-house |
| 22Rv1 | Zwart lab, in-house |
| PreB cells | In-house |
| Mouse Embryonic Fibroblasts | In-house |
| HCT116, P53-wt/ko | Wang *et al.*^20^ |
| MCF-7, P53-wt/ko | Wellenstein *et al.*^19^ |
| A375 | Bernard’s Lab, in-house |
| SKOV3 | Bernard’s Lab, in-house |
| HEK 293T cells | Jonker’s Lab, in-house |
| J558-IL-7 producer cells | Rolink (2) |

**Table S5:** Vectors used in this study.

| **Recombinant DNA** | **Source** |
| --- | --- |
| pCL-ECO | In-house, see^25^ |
| pMX-IRES-GFP | In-house, see^25^ |
| pMX-mPOLK-GFP | In-house, see^25^ |
| pX330-puro | In house, from Henri |
| pX333-mCherry | In-house, see^18^ |
| pMX-eGFP | Amaxa, Lonza |

**Table S6:** Reagents used in this study.

| **Reagents** | **Source** |
| --- | --- |
| JH-RE-06 (REV1 inhibitor) | MedChem express; cat HY-126214 |
| Cisplatin | In-house |
| RNAImax | Thermofisher; cat 13778030 |
| Nucleofector Cuvettes | Sopachem BV, mirus; cat. MIR 50121 |

**Table S7:** Antibodies used in this study.

| **Antibodies used in study** | **Manufacturer** | **Dilution** |
| --- | --- | --- |
| cKit-APC, 2B8 | eBioscience | 1:200 |
| Streptavidin-APC-Cy7 | Southern Biotech | 1:200 |
| CD135-APE, A2F10 | Biolegend | 1:200 |
| CD48-PE-Dazzle, HM48-1 | Biolegend | 1:200 |
| 7AAD-PE-Cy5 | Biolegend | 1:200 |
| Sca-1-PE-Cy7, D7 | Biolegend | 1:200 |
| CD34-FITC, RAM34 | Biolegend | 1:100 |
| CD127-BV421, A7R34 | Biolegend | 1:200 |
| CD150-BV650, TC15-12F12.2 | Biolegend | 1:200 |
| CD16/32-BV785, 2.4G2 | BD Bioscience | 1:200 |
| Lineage cell detection cocktail biotin | Miltenyi | 1:40 |
| UltraComp eBeads | Invitrogen | 1 drop/5 samples |
| Rev1, OTIE12 | Invitrogen | 1:200-1:500 |
| GFP, 3E6 | Invitrogen | 1:1000 |
| ß-Actin, AC-15 | Sigma Aldrich | 1:5000 |
| PCNA (PC-10) | Santa Cruz | 1:1000 |
| Actin, clone C4 | Merck Milipore | 1:10000 |
| FLAG monoclonal, F3165 | Sigma-Aldrich | 1:1000 |
| HorseRadish | Dako | 1:2500 |
| Horse Radish Peroxidase | Dako | 1:2500 |
| 680-RD, anti-mouse | LiCor | 1:10000 |
| 800-CW, anti-mouse | LiCor | 1:10000 |
| PNA-FITC | Vector Laboratories | 1:400 |
| CD19-APC, 1D3 | BD pharmingen | 1:300 |
| CD95-PE, Jo2 | BD pharmingen | 1:200 |
| CD43-Biotin, S7 | BD biosciences | 1:100 |

**Table S8:** PCR settings

| **PCR settings** | **Time** |
| --- | --- |
| 95ºC | 3min |
| 75ºC | 5min |
| 72ºC | 1.5min |
| Melting - 95ºC (30-40 cycles) | 30sec |
| Annealing - 63ºC (30-40 cycles) | 30sec |
| Extension - 72ºC (30-40 cycles) | 45sec |
| Final extension - 72ºC | 10min |
| Storage – 4ºC-12ºC | Until usage |
